# High bilateral bony symmetry in dysplastic and nondysplastic knees: a CT-based 3D evaluation

**DOI:** 10.1186/s12891-025-09272-w

**Published:** 2025-12-29

**Authors:** Miriam R. Boot, Sebastiaan A.W. van de Groes, Julia van den Heiligenberg, Esther Tanck, Dennis Janssen

**Affiliations:** 1 Orthopaedic Research Laboratory, Department of Orthopaedics, Radboud university medical center, Grooteplein Zuid 10, Nijmegen, 6525 GA The Netherlands; 2 Technical Medicine, University of Twente, Hallenweg 5, Enschede, 7522 NH The Netherlands

**Keywords:** Bilateral asymmetry, Trochlear dysplasia, Knee morphology, CT scans, Side-to-side comparison, 3D analysis

## Abstract

**Background:**

Side-to-side comparisons of knee joints are commonly used in orthopaedic research and surgery because natural pairs provide strong internal control for genetic, developmental, and environmental factors. This approach generally assumes that the contralateral knee represents a healthy anatomical baseline. However, it remains unclear whether this assumption holds in the presence of trochlear dysplasia, a key risk factor for patellofemoral instability (PFI), as dysplasia may affect the degree of bilateral symmetry in knee morphology. This study aimed to quantify three-dimensional (3D) morphological bony symmetry of knee joints and evaluate the impact of trochlear dysplasia.

**Methods:**

Bilateral Computed tomography (CT) scans from 187 subjects were analysed, including 137 without trochlear dysplasia, 37 with low-grade dysplasia, and 13 with high-grade dysplasia. Surface models of the distal femur, patella, and proximal tibia were generated using automated segmentation with manual refinement. Correspondence points (CPs) were established via rigid registration of mirrored left-side models to right-side counterparts, followed by non-rigid registration of the right-side models onto the aligned left-side models. Symmetry was quantified as Euclidean distances between CPs.

**Results:**

Median CP distances were 0.8 mm in nondysplastic femurs and tibiae (1st–99th percentile 0.2–2.0/2.1 mm) and 0.9 mm in dysplastic femurs and tibiae (1st–99th percentile 0.2–2.1/2.3 mm), indicating a high degree of bilateral symmetry. Localized asymmetries of approximately 2–3 mm were observed, although no consistent anatomical patterns were identified. Patellar asymmetry increased with dysplasia severity, although it was primarily due to loose bone fragments rather than true anatomical variation.

**Conclusions:**

Both nondysplastic and dysplastic knees exhibit high bilateral bony symmetry. Although CP distances were slightly higher in dysplastic knees, femoral and tibial CP distances remained small (group-level 99th percentiles ≤ 2.3 mm) and are therefore likely clinically irrelevant.

**Supplementary Information:**

The online version contains supplementary material available at 10.1186/s12891-025-09272-w.

## Background

Accurate assessment of knee joint morphology is essential for orthopaedic surgical planning and biomechanical modelling, because natural pairs provide strong internal control for genetic, developmental, and environmental factors. In unilateral joint pathologies, the contralateral knee is often used as a reference to evaluate anatomical deviations or to guide corrective procedures [1]. This practice relies on the assumption that the contralateral knee represents a healthy anatomical baseline, and that any asymmetry reflects pathological change.

In healthy populations, this assumption is generally supported, as studies have shown a high degree of bilateral knee symmetry [2–4]. However, its validity in patients with patellofemoral instability (PFI) remains uncertain. Chen et al. [5] highlighted bilateral morphological asymmetry in patients with unilateral PFI, with affected knees showing more pronounced trochlear dysplasia, greater tibial tubercle-trochlear groove (TT-TG) distances and reduced lateral trochlear inclination (LTI). Although contralateral knees also showed abnormalities, their milder severity led the authors to propose them as a potential template for osteotomy planning. Conversely, Demehri et al. [6] emphasized that asymptomatic contralateral knees in patients with unilateral PFI demonstrated morphological differences compared to healthy controls, including a shallower trochlear groove, increased TT-TG distance, and imaging features of early osteoarthritis. Together, these studies suggest that contralateral knees in PFI patients are not truly normal, although the extent of their abnormalities and their clinical relevance remain uncertain. Trochlear dysplasia, the main anatomical risk factor for PFI, has a prevalence of 35.7–96% among patients compared to only 3–4% in the general population [7], making it a robust basis for studying bilateral asymmetry. Its quantification on imaging offers a more objective assessment than self-reported or retrospective evaluations of unilateral or bilateral PFI, which are more prone to underestimation or bias [8]. Examining bilateral symmetry across different severities of trochlear dysplasia could therefore provide valuable insights into the nature of anatomical asymmetry in PFI.

Previous studies have primarily relied on two-dimensional (2D) radiographic measurements such as LTI and TT-TG distance [5, 6]. However, these measurements are susceptible to slice selection bias and misalignment errors [9, 10]. In contrast, three-dimensional (3D) morphological analysis captures the full geometry of the distal femur, patella, and proximal tibia, providing a more comprehensive and reproducible evaluation of bony symmetry.

The aim of this study was to evaluate bilateral bony symmetry of the distal femur, patella, and proximal tibia using 3D morphological analysis, and to determine whether the degree of symmetry differs between knees with low- and high-grade trochlear dysplasia compared to nondysplastic knees. We hypothesized that both dysplastic and nondysplastic knees would demonstrate a high degree of bilateral bony symmetry without clinically relevant differences.

## Methods

### CT scan selection criteria and dataset composition

Knee symmetry was assessed using computed tomography (CT) scans performed at the Radboud university medical center. Scans were included if they met the following inclusion criteria: voxel size ≤ 1.0 mm in all directions, visibility of the knee joint (including the trochlea, the entire patella, and the tibial tuberosity), fused growth plates, and absence of visible motion artifacts. Scans were excluded if there was evidence of a bipartite patella, patellar luxation in extension (suggesting chronic instability), signs of prior surgery, or any known structural, neoplastic, developmental, or post-traumatic abnormalities that could affect knee anatomy.

A total of 187 CT scans met these criteria. Of these, 98 were sourced from a dataset of healthy volunteers aged 18 to 35 years [11]. The study was approved by the Institutional Review Board, and all participants provided written informed consent for secondary use of their data prior to participation according to the Declaration of Helsinki. These CT scans were acquired between November 2020 and June 2021 using a 320-channel wide-area CT scanner (Aquilion ONE, Canon Medical Systems, Otawara, Japan), with an average voxel size of 0.71 × 0.71 × 0.80 mm, and a scan range of 50.0 cm. The remaining 89 CT scans were collected from the electronic health records system and included patients aged 16 to 40 years at the time of scanning, performed between November 2013 and February 2025. No ethical approval was required for this retrospective data, as confirmed by the local ethics committee. The average voxel size was 0.72 × 0.72 × 0.64 mm, with an average scan range of 40.5 cm.

### Trochlea dysplasia classification

Trochlear dysplasia was classified by an experienced orthopaedic surgeon specializing in knee surgery (SvdG) using the Dejour classification system [12]. Based on the severity of dysplasia, knees were categorized into three groups: no dysplasia, low-grade dysplasia (Dejour type A), and high-grade dysplasia (Dejour types B–D). This simplified two-grade system was chosen to avoid overly small subgroup sizes and because of improved reliability compared to the four-grade system [13]. Classification was based on lateral knee radiographs when available. In cases where radiographs were not available (170 of 187 pairs), classification was performed using digitally reconstructed radiographs derived from CT scans, with direct CT evaluation used as additional support when necessary. For knee pairs with asymmetrical dysplasia grading, the more severe classification was assigned to the pair. Following classification, 137 knee pairs were identified as having no trochlear dysplasia, 37 as low-grade, and 13 as high-grade. Subject demographics for each group are summarized in Table 1. Medical histories for these subjects were not available.

**Table 1:** Subject demographics by trochlear dysplasia classification

Category	No trochlear dysplasia (*n* = 137)	Low-grade trochlear dysplasia (*n* = 37)	High-grade trochlear dysplasia (*n* = 13)
Age (years), mean ± SD (range)	24.1 ± 4.6 (16–38)	22.3 ± 4.6 (16–33)	21.3 ± 5.8 (16–34)
Sex (Male: Female), n	51:86	11:26	4:9

*SD* standard deviation

### Surface model creation

The femur, patella, and tibia of both knees were segmented from the CT scans using an nnUNet deep learning model trained on a healthy knee dataset (Dice similarity coefficients: femur 0.99, patella 0.97, tibia 0.98) [14]. The initial segmentations were manually refined in 3D Slicer (version 5.0.3, Brigham and Women’s Hospital, Boston, MA, USA) to ensure anatomical accuracy. Following refinement, segmentations were converted into 3D surface models in MATLAB (The MathWorks Inc., Natick, MA, USA). To ensure consistent model resolution, surface meshes were remeshed in Hypermesh (Altair Engineering Inc., Troy, MI, USA) to achieve uniform element sizes of 2.0 mm. The fabella and any loose bone fragments, such as fragments resulting from patellar avulsion fractures, were excluded during segmentation.

### Morphological symmetry evaluation

**Fig. 1:**
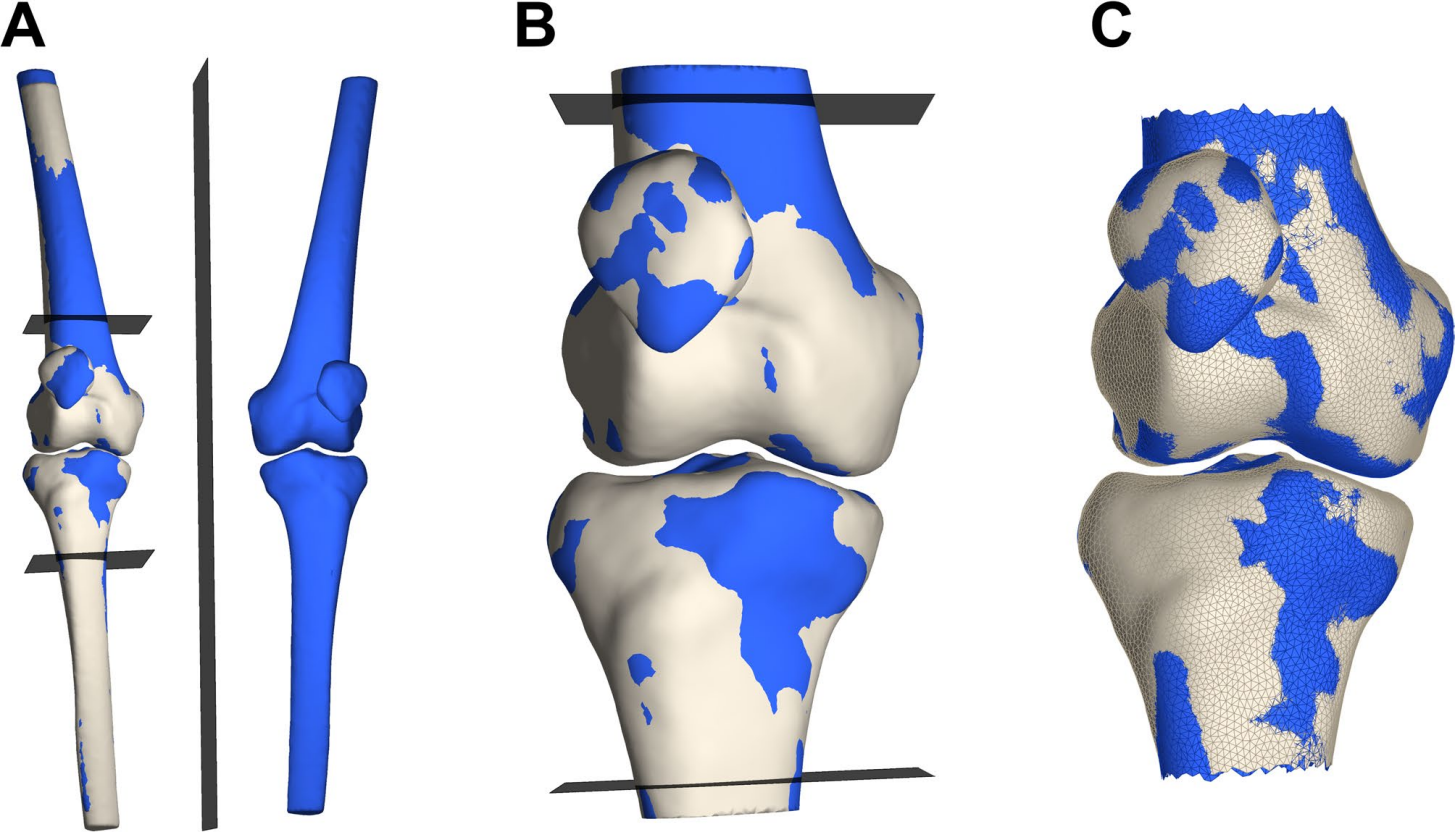
Workflow for evaluating morphological symmetry of a knee pair. (**A**) Left femur, patella, and tibia models (blue) were mirrored in the sagittal plane and rigidly registered to their right-side counterparts (beige) using Coherent Point Drift surface matching. (**B**) Femur and tibia models were trimmed in the proximal-distal direction to derive surface models with heights matching the medial-lateral width. (**C**) Correspondence points within 2.0 mm of the cutting planes were excluded, and point-to-point Euclidean distances were calculated to quantify morphological symmetry

Morphological symmetry between left and right knees was assessed through 3D surface model comparison, based on a previously described method [15] (Fig. 1). First, the left-side femur, patella, and tibia models were mirrored in the sagittal plane and rigidly registered to their contralateral right-side counterparts using Coherent Point Drift (CPD) surface matching [16]. Each bone was aligned separately, independent of its anatomical position within the knee. To focus the analysis on joint-relevant morphology, the femur and tibia models were trimmed in the proximal-distal direction. This trimming required alignment with the global coordinate system, which was based on the anatomical coordinate system of each individual bone for sufficiently long models [17–19], and via registration to a pre-aligned reference model for shorter models. This alignment was followed by two iterations of trimming and rigid registration to achieve model heights matching the medial-lateral range of the surface mesh or the maximum available height minus 5 mm for shorter bones, ensuring straight and symmetrical cuts. Left patella models were registered directly to their right-side counterparts without trimming. After rigid alignment, nonrigid CPD surface matching was applied to deform the right-side models to best fit the mirrored and rigidly aligned left-side models, allowing calculation of point-to-point Euclidean distances between corresponding nodes. To avoid artificial asymmetries caused by residual trimming differences, correspondence points (CPs) within 2.0 mm of the femoral and tibial cutting planes were excluded. Symmetry was evaluated at a median of 7,224 CPs for the femur (interquartile range (IQR), 6,804–8,069), 1,721 CPs for the patella (IQR, 1,596–1,890), and 5,681 CPs for the tibia (IQR, 5,341–6,450).

### Statistical analysis

Morphological symmetry data were assessed for normality using histograms, which revealed a non-normal distribution. Therefore, results are reported as medians with IQRs. Boxplots were used to illustrate overall symmetry distributions for each bone, while heatmaps were generated to visualize regional asymmetries. Due to the large number of CP measurements per bone, even clinically irrelevant differences could reach statistical significance, increasing the risk of Type I errors. Therefore, no formal testing was performed. All analyses were conducted using MATLAB.

## Results

**Fig. 2:**
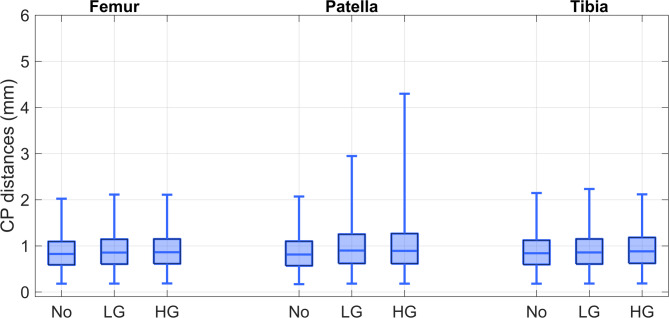
Boxplot showing morphological symmetry of the distal femur, patella, and proximal tibia models in knee pairs with no (No, *n* = 137), low-grade (LG, *n* = 37), and high-grade (HG, *n* = 13) trochlear dysplasia. Symmetry is expressed as Euclidean distances between left and right correspondence points (CPs). Each box represents 2.4–10.2 × 10⁵ CPs (no dysplasia), 6.2–26.9 × 10⁴ CPs (low-grade), and 2.2–. 9.6 × 10⁴ (high-grade). Whiskers denote the 1 st to 99th percentiles. Please refer to Supplementary Material 1–3 for boxplots showing morphological symmetry per knee pair

**Fig. 3:**
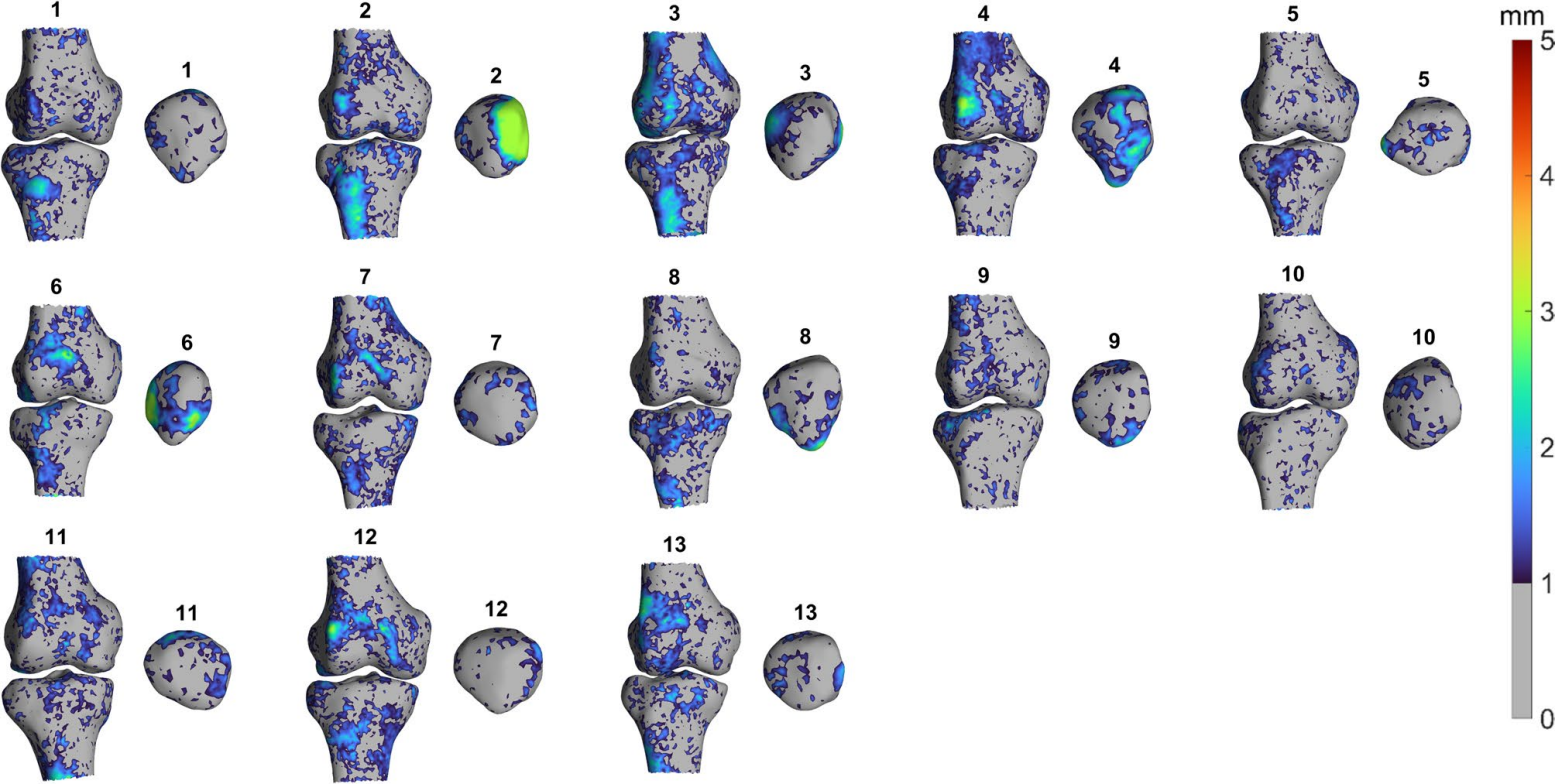
Anterior heatmaps showing left-right morphological differences of all 13 knee pairs with high-grade dysplasia, displayed on the right knees. CP distances ≤ 1.0 mm are shown in grey, reflecting the measurement accuracy threshold. Please refer to Supplementary Material 4–6 for the corresponding posterior heatmaps and for heatmaps of the low-grade and no dysplasia groups

**Fig. 4:**
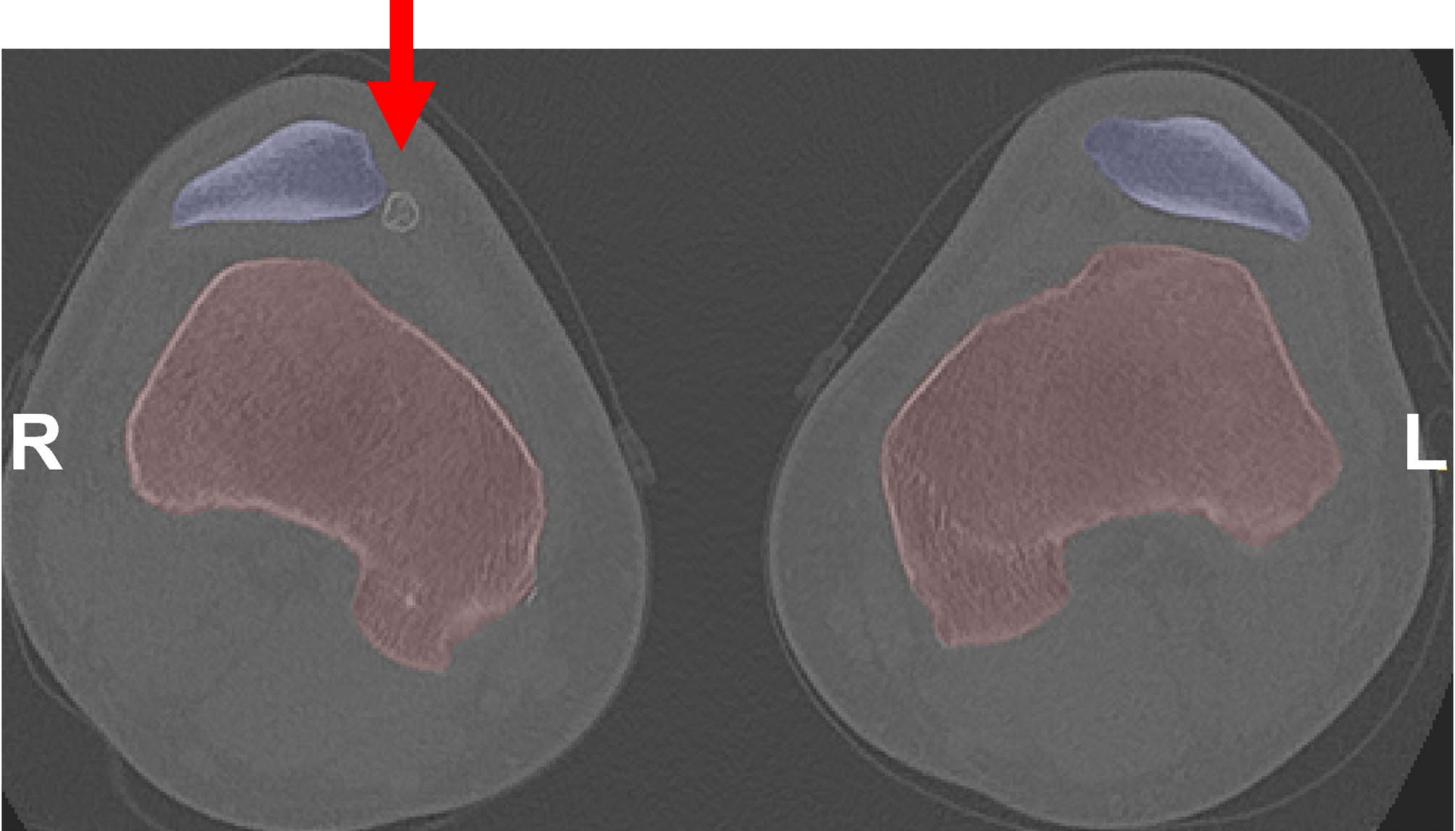
Axial CT slice of a knee pair with high-grade dysplasia (knee pair 2), demonstrating marked asymmetry in the patella (see Fig. 3, number 2). The asymmetry is attributed to a loose patellar fragment, indicated by the red arrow

Morphological symmetry of the distal femur, patella, and proximal tibia was quantified by calculating Euclidean distances between CPs on mirrored left and right knee models (Fig. 2; Table 2). In knees without trochlear dysplasia, median CP distances were highly consistent across the femur, patella, and tibia, with a median of 0.8 mm (IQR: 0.6–1.1 mm; 1st–99th percentile: 0.2–2.0/2.1 mm), indicating a high degree of bilateral symmetry. In knees with low- or high-grade dysplasia, femoral and tibial CP distances increased only slightly, to a median of 0.9 mm (IQR: 0.6–1.1/1.2 mm; 1st–99th percentile: 0.2–2.1/2.3 mm), suggesting that overall symmetry was largely preserved. Variability in patellar asymmetry increased with dysplasia severity, as shown by wider 1st–99th percentile ranges (low-grade dysplasia: 0.2–2.9 mm; high-grade dysplasia: 0.2–4.3 mm). Heatmaps and the corresponding CT scans revealed that these asymmetries were primarily associated with patellae containing loose bone fragments, which were frequently present in high-grade dysplasia (> 50% of knees) but rare in nondysplastic knees (~1%) (Figs. 3 and 4). Although these fragments were excluded from the segmentations, they altered the apparent patellar shape and influenced perceived symmetry between sides.

**Table 2:** Left-right morphological differences (mm) across the distal femur, patella, and proximal tibia in knee pairs with no, low-grade, and high-grade trochlear dysplasia. Values are median correspondence point (CP) distances with interquartile ranges (IQRs). Statistical testing was omitted because the large number of CP measurements per bone could lead to statistically significant but clinically irrelevant differences, increasing the risk of type I errors

	Morphological bone symmetry		
	Femur	Patella	Tibia
	Correspondence point distances (mm)		
No trochlear dysplasia (*n* = 137)	0.8 (0.6–1.1)	0.8 (0.6–1.1)	0.8 (0.6–1.1)
Low-grade trochlear dysplasia (*n* = 37)	0.9 (0.6–1.1)	0.9 (0.6–1.3)	0.9 (0.6–1.2)
High-grade trochlear dysplasia (*n* = 13)	0.9 (0.6–1.2)	0.9 (0.6–1.3)	0.9 (0.6–1.2)

No other regions seemed to show consistent asymmetry across the cohort based on visual inspection, indicating the absence of systematic anatomical deviation. Localized asymmetries in the femur and tibia that exceeded the 1.0 mm accuracy threshold were spread around, reaching approximately 2–3 mm in both dysplastic and nondysplastic knees (Figs. 3, Supplementary Material 4–6).

## Discussion

The most important findings of this study were that both nondysplastic and dysplastic femurs and tibiae exhibit a high degree of bilateral bony symmetry, reflected by consistently low median and 1st–99th percentile CP distances (nondysplastic: median 0.8 mm, 1st–99th percentile 0.2–2.0/2.1 mm; dysplastic: median 0.9 mm, 1st–99th percentile 0.2–2.1/2.3 mm). These small left-right differences are approximately equal to the spatial resolution limits of CT imaging and align closely with the findings of Jang et al. [4], who reported average 3D alignment deviations of 0.8 mm for the distal femur and 1.1 mm for the proximal tibia in healthy cadaveric knees. Although patellar asymmetry increased with dysplasia severity, this was largely attributable to loose patellar fragments, likely resulting from avulsion fractures due to prior dislocations [20], which contributed to measured asymmetries without reflecting true anatomical variation.

Our findings of high bilateral bony symmetry in dysplastic femurs and tibiae contrast with those of Chen et al. [5], who concluded that ipsilateral knees in patients with unilateral PFI exhibit more severe morphological abnormalities than those of contralateral knees. Several factors may explain this discrepancy. Most notably, their cohort consisted of patients with confirmed unilateral PFI, whereas ours was stratified by trochlear dysplasia severity regardless of PFI history. However, based on our results, it is also plausible that knee asymmetry is not clinically relevant, even in unilateral PFI cases. First, the significant differences they reported using the four-grade Dejour classification may be influenced by the limited inter-observer reliability of this system [13]. Second, their reported left–right differences in LTI (mean: 1.8°) were small, and the corresponding morphological differences may have fallen within or near our estimated measurement accuracy (~ 1.0 mm). Similarly, their TT–TG differences (mean: 1.9 mm) may reflect alignment variation rather than true morphological asymmetry, given that 2D CT measurements are susceptible to leg positioning errors [9, 10], with TT–TG distance varying by approximately 0.5 mm per degree of tibial rotation [10]. Future studies combining 3D morphological assessment with confirmed PFI status are warranted to clarify the extent and clinical significance of side-to-side asymmetries in these patients.

In our cohort, the 99th percentile CP distances for femurs and tibiae across all dysplasia severities ranged from 2.0 to 2.3 mm at group level (Fig. 3). These values are close to the error margins of patient-specific instruments and 3D surgical guides (≈ 2 mm) [21–23], suggesting that the observed asymmetries are likely clinically irrelevant. Therefore, this supports the validity of side-to-side comparisons in orthopaedic assessments, particularly in posttraumatic conditions. However, the high degree of symmetry also indicates that contralateral knees may not reliably serve as surrogate ‘healthy’ templates for correction of congenital abnormalities such as trochleoplasty or tibial tubercle osteotomy. Moreover, the high bilateral similarity of the femoral trochlea within the same individual suggests that trochlear dysplasia alone may not fully explain the development of recurrent PFI. Beyond these clinical considerations, our findings contribute to the etiological debate on trochlear dysplasia. The overall symmetry across dysplasia severities largely supports a constitutional origin [12, 24, 25], while subtle asymmetries in some dysplastic pairs may reflect developmental influences, such as altered patellar tracking during growth [26–28].

Several limitations should be recognized. First, although scans were excluded based on CT indications and visible conditions (e.g., prior surgery), full medical histories were unavailable. Consequently, we could not rule out the presence of other undetected conditions that may have contributed to localized asymmetries observed in a few nondysplastic and dysplastic knees (Supplementary Material 1–3). Additionally, it remains unclear whether subjects in the low- and high-grade dysplasia groups experienced PFI. Future studies should replicate this analysis in cohorts with confirmed PFI. Second, the relatively small sample size in the high-grade dysplasia group of 13 knee pairs limits the generalizability of our findings. Nonetheless, the inclusion of this group provides valuable preliminary insights into 3D symmetry patterns in dysplastic knees. Third, our assessment of symmetry was limited to visual inspection and descriptive statistics of CP distances per bone. We did not perform region-specific symmetry analysis or apply formal statistical testing, which may limit the ability to detect subtle or localized asymmetries. Nevertheless, this study provides an initial indication of the absence of clinically relevant asymmetry in dysplastic knees. Future studies should validate these findings using detailed regional analysis and appropriate statistical methods. Finally, the accuracy of left-right comparisons was limited by the spatial resolution of the CT scans and potential segmentation errors, particularly in low-contrast regions such as tibial baseplate edges, where distinguishing bone from background was sometimes difficult. To reduce these effects, scans with voxel dimensions exceeding 1.0 mm were excluded. Despite these limitations, our approach enabled a comprehensive 3D evaluation of knee morphology in a large dataset, offering a more anatomically detailed and reproducible assessment of bilateral symmetry than traditional 2D methods. To date, evidence on 3D morphological symmetry of the femur, patella, and tibia across varying severities of trochlear dysplasia has been limited.

## Conclusions

This study demonstrates that both nondysplastic and dysplastic femurs and tibiae exhibit a high degree of bilateral bony symmetry, with median left-right differences close to the spatial resolution limits of CT imaging. Although patellar asymmetry increased in dysplastic knees, this was primarily due to loose fragments and did not reflect true anatomical variation. These findings support the continued use of contralateral knees as anatomical references in clinical and research settings. However, in congenital abnormalities such as trochlear dysplasia, contralateral comparison should be interpreted with caution, as high bilateral symmetry may limit its utility as a corrective template.

## Supplementary Information

Supplementary Material 1.

## Data Availability

The CT scan dataset analysed during the current study are available from the corresponding author on reasonable request. Other data used in this study are available at 10.34973/2vbq-yv30.

## Abbreviations

CP: Correspondence point
CPD: Coherent Point Drift
CT: Computed tomography
IQR: Interquartile range
LTI: Lateral trochlear inclination
PFI: Patellofemoral instability
3D: Three-dimensional
TT-TG: Tibial tubercle-trochlear groove
2D: Two-dimensional
