# Supplementary material for: High bilateral bony symmetry in dysplastic and nondysplastic knees: a CT-based 3D evaluation
Source: BMC Musculoskelet Disord. 2025 Dec 29;26:1109. doi: 10.1186/s12891-025-09272-w (PMC12750923; doi:10.1186/s12891-025-09272-w)

**Supplementary material**

| **Supplementary Material 1** | Boxplots showing the morphological bony symmetry of the distal femur, patella, and proximal tibia models of knee pairs with no trochlear dysplasia, with each box representing a bone pair. |
| --- | --- |
| **Supplementary Material 2** | Boxplots showing the morphological bony symmetry of the distal femur, patella, and proximal tibia models of knee pairs with low-grade trochlear dysplasia, with each box representing a bone pair. |
| **Supplementary Material 3** | Boxplots showing the morphological bony symmetry of the distal femur, patella, and proximal tibia models of knee pairs with high-grade trochlear dysplasia, with each box representing a bone pair. |
| **Supplementary Material 4** | Heatmaps illustrating anterior and posterior left-right differences in bone morphology for knee pairs with no trochlear dysplasia. |
| **Supplementary Material 5** | Heatmaps illustrating anterior and posterior left-right differences in bone morphology for knee pairs with low-grade trochlear dysplasia. |
| **Supplementary Material 6** | Heatmaps illustrating anterior and posterior left-right differences in bone morphology for knee pairs with high-grade trochlear dysplasia. |

| **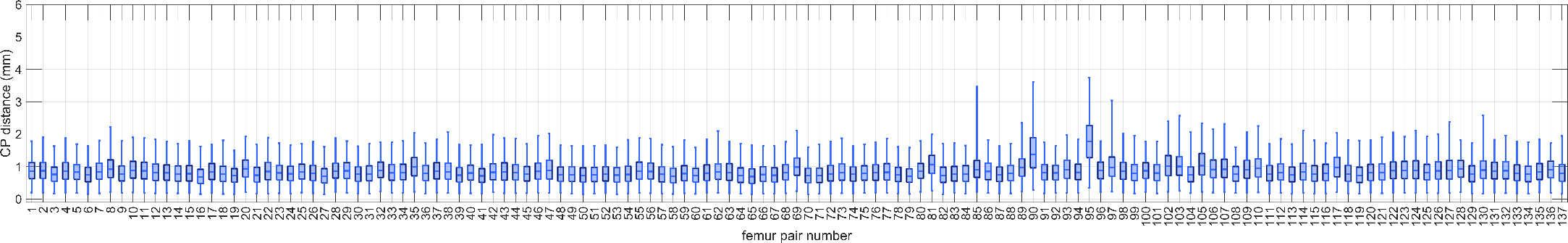** |
| --- |
| **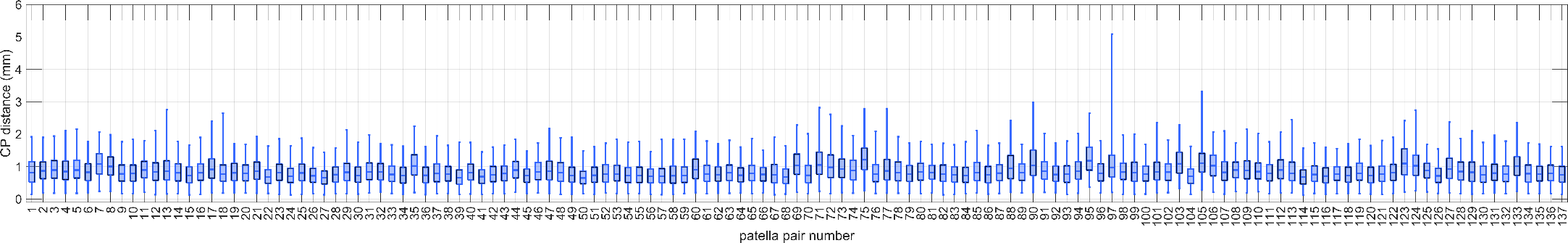** |
| **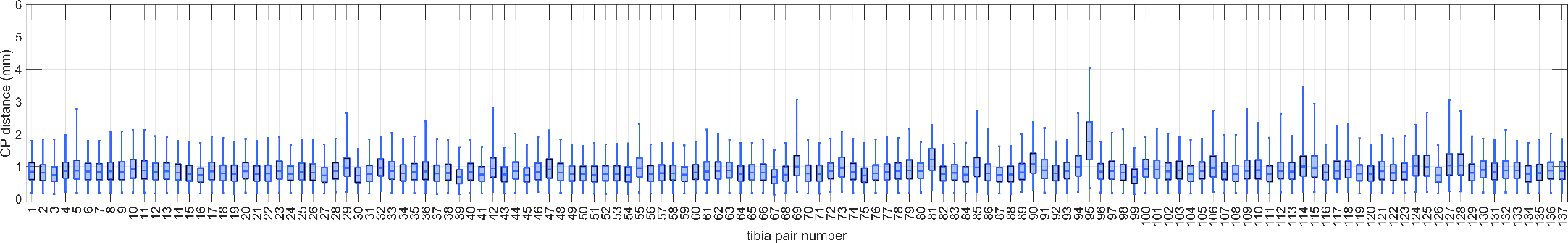** |
| **Supplementary Material 1** Boxplots showing the morphological bony symmetry of the distal femur, patella, and proximal tibia models of knee pairs with no trochlear dysplasia, with each box representing a bone pair. Morphological symmetry is quantified as Euclidean distance between the correspondence points (CPs). The whiskers represent the 1st to the 99th percentile. |

| 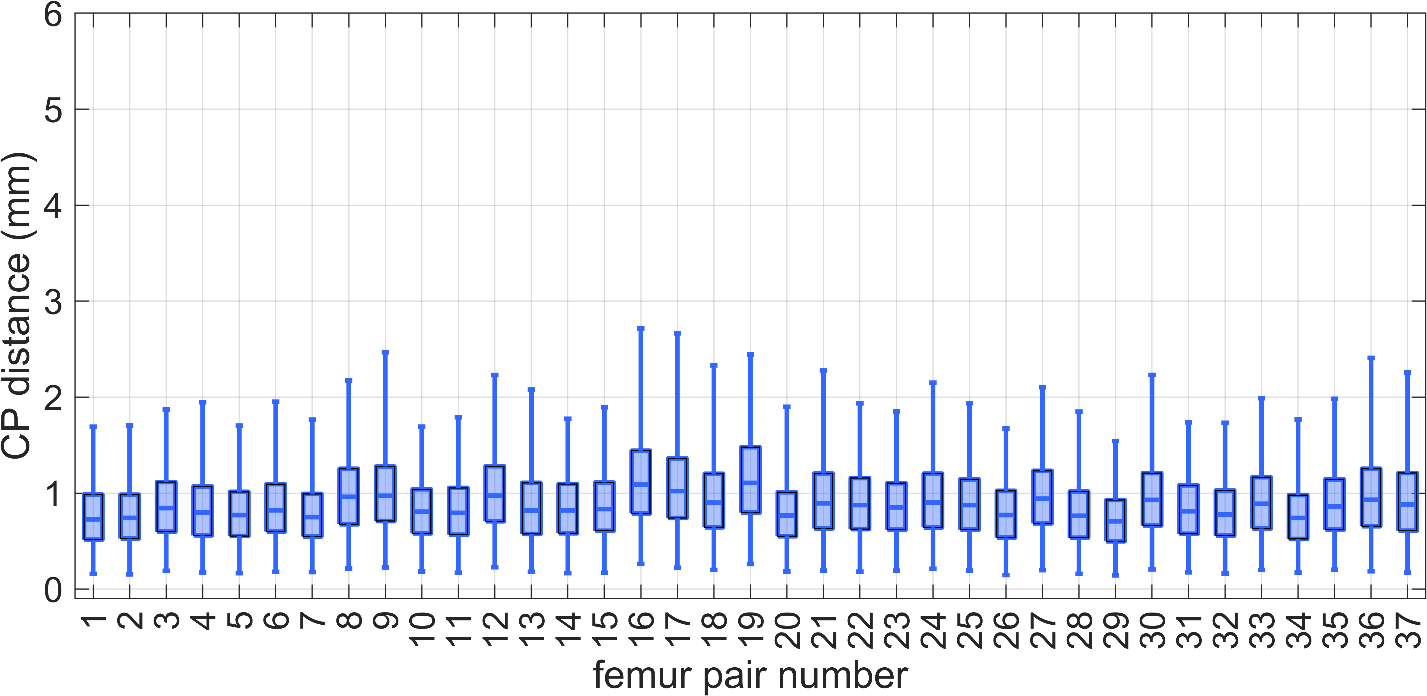 |
| --- |
| 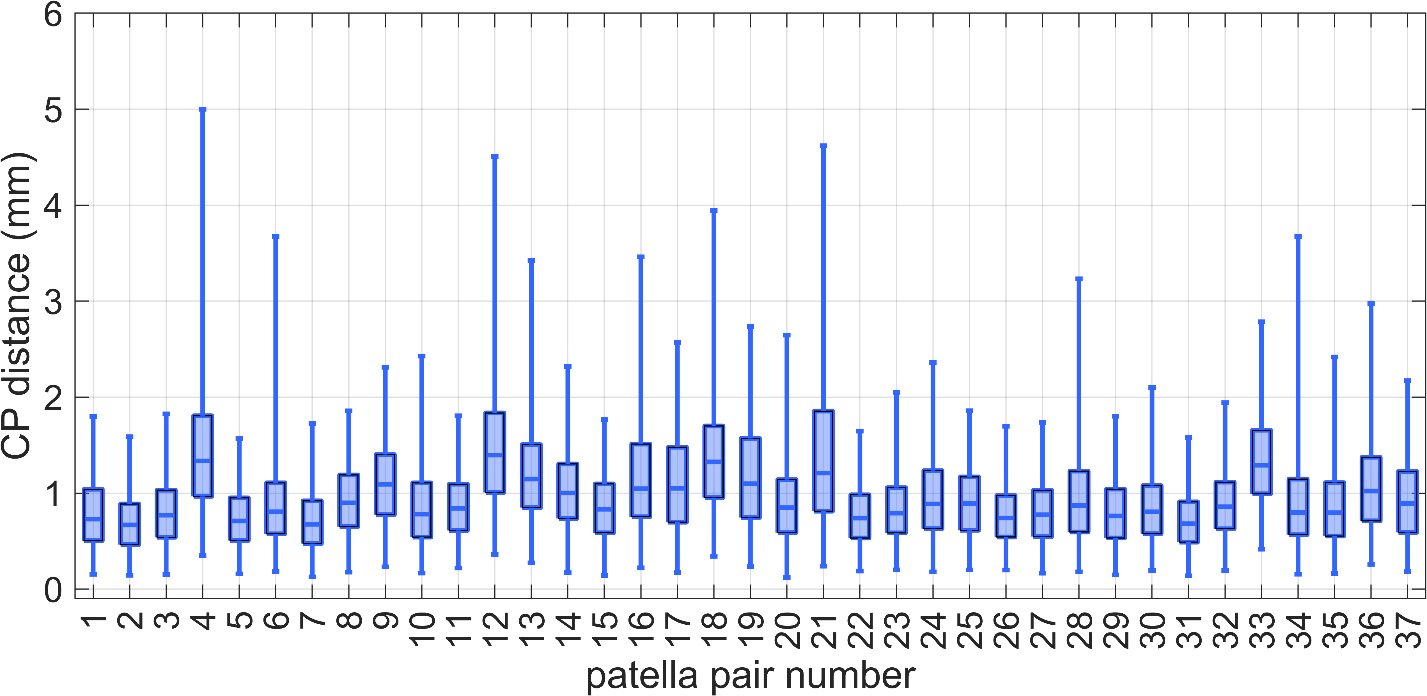 |
| 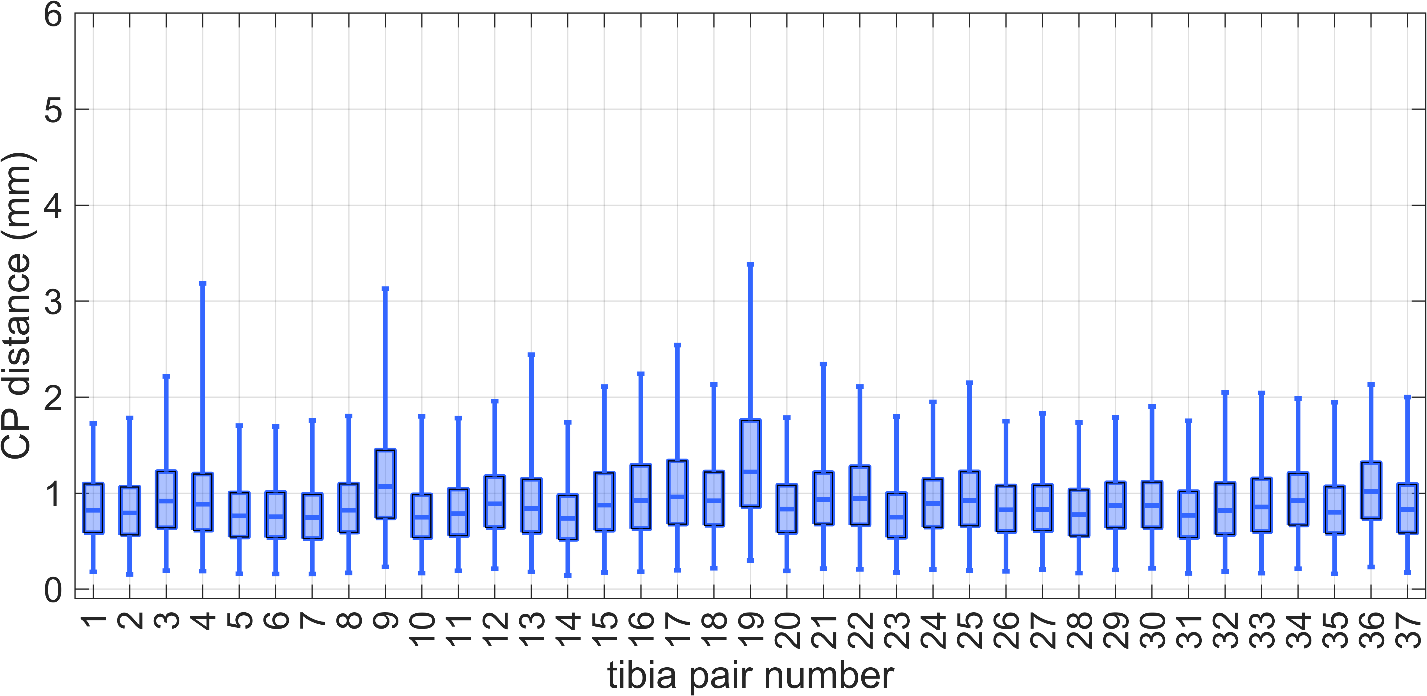 |
| **Supplementary Material 2** Boxplots showing the morphological bony symmetry of the distal femur, patella, and proximal tibia models of knee pairs with low-grade trochlear dysplasia, with each box representing a bone pair. Morphological symmetry is quantified as Euclidean distance between the correspondence points (CPs). The whiskers represent the 1st to the 99th percentile. |

| **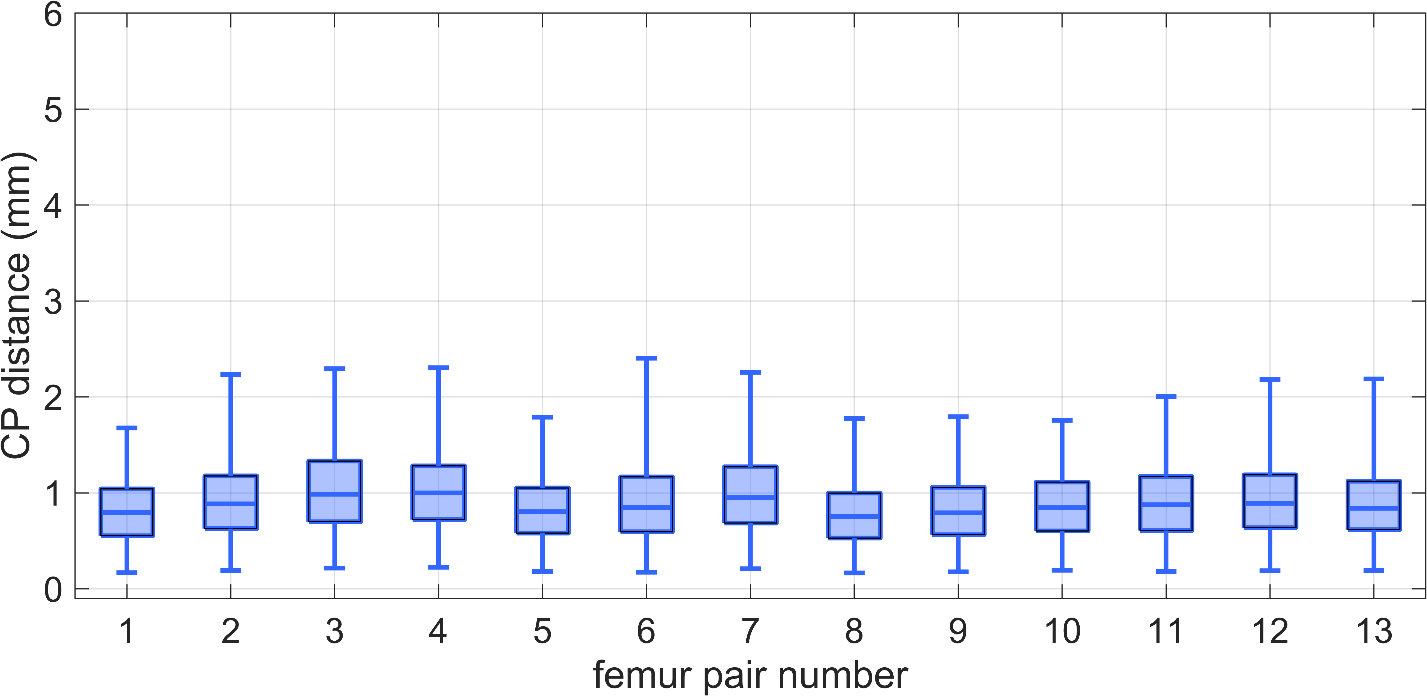** |
| --- |
| **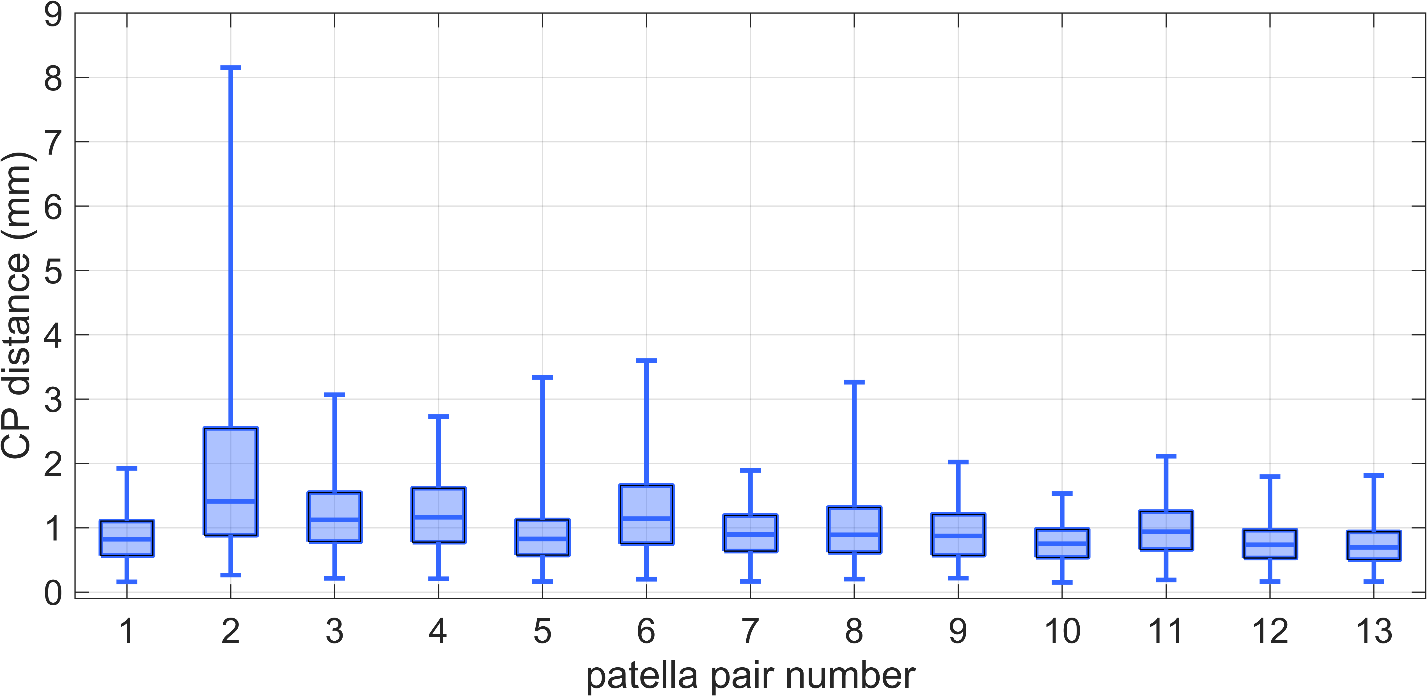** |
| **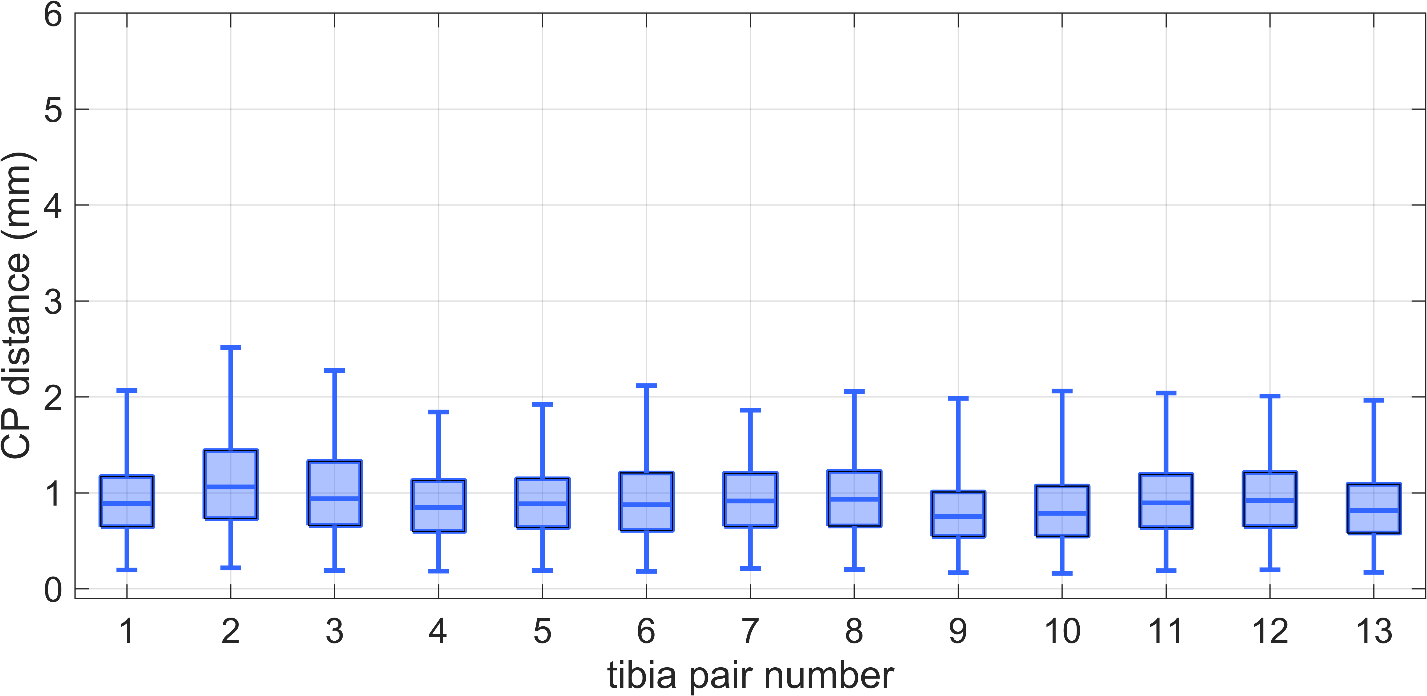** |
| **Supplementary Material 3** Boxplots showing the morphological bony symmetry of the distal femur, patella, and proximal tibia models of knee pairs with high-grade trochlear dysplasia, with each box representing a bone pair. Morphological symmetry is quantified as Euclidean distance between the correspondence points (CPs). The whiskers represent the 1st to the 99th percentile. |

| 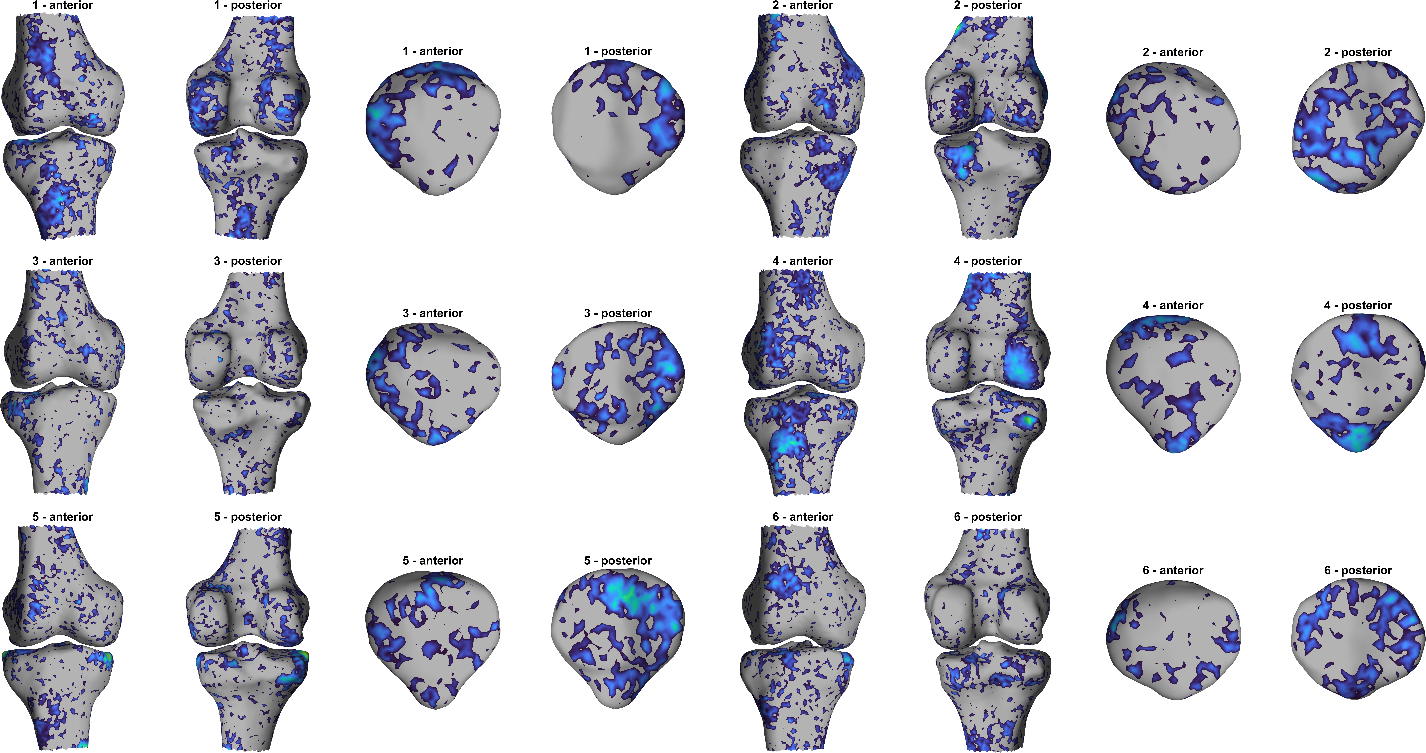  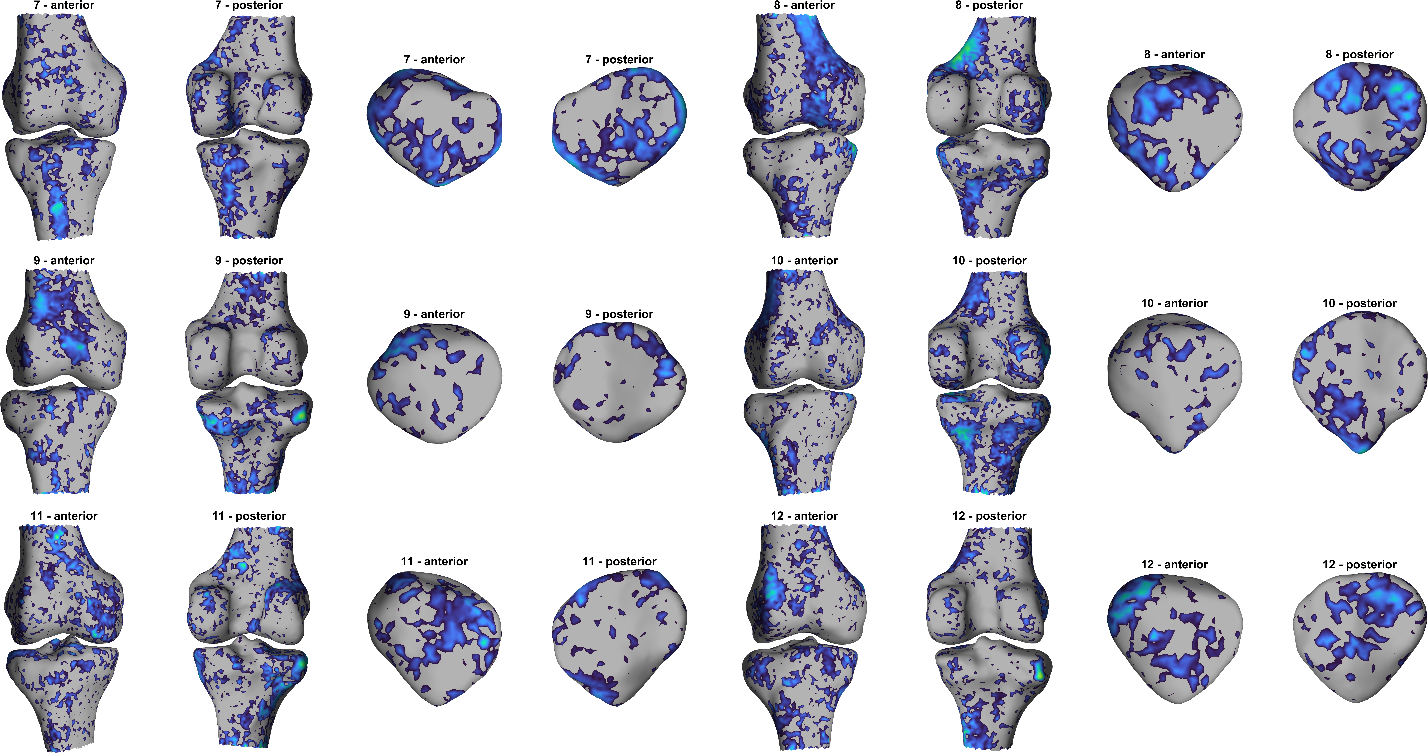  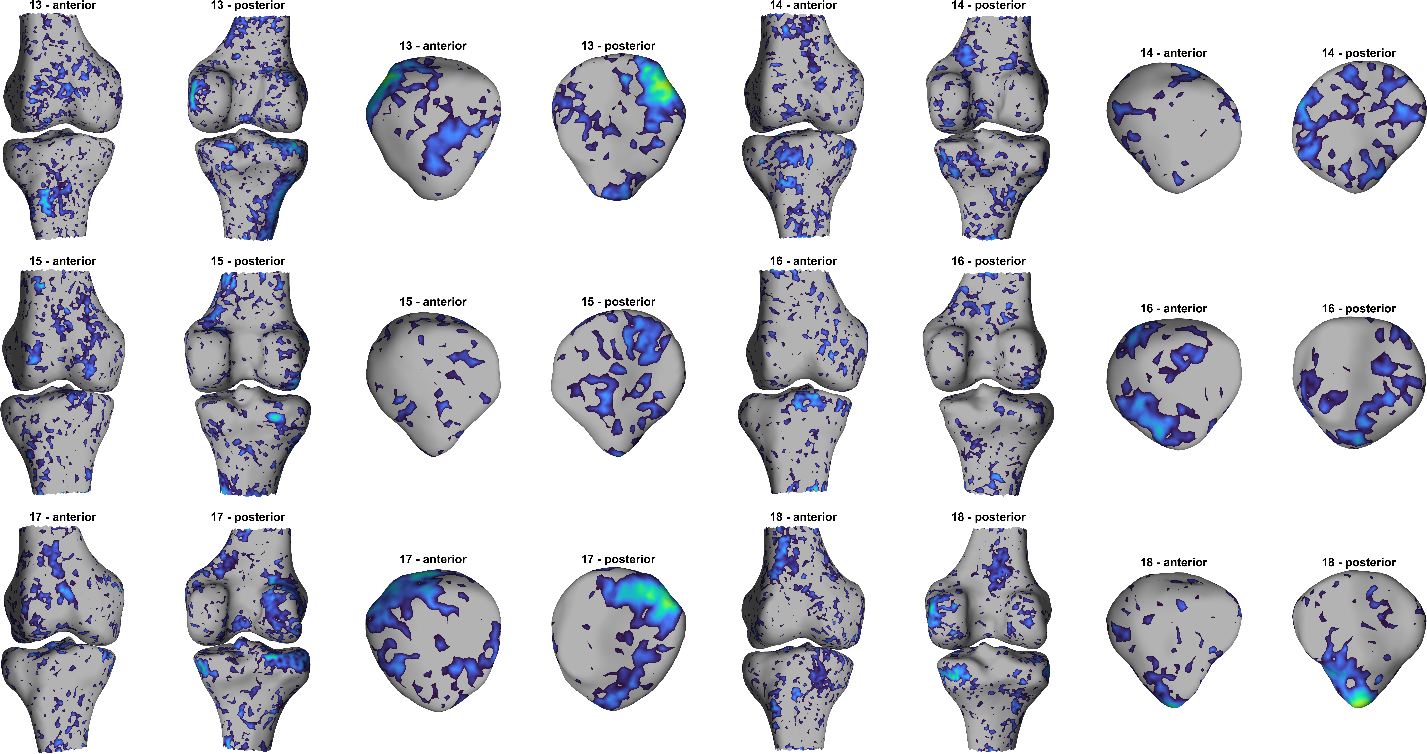  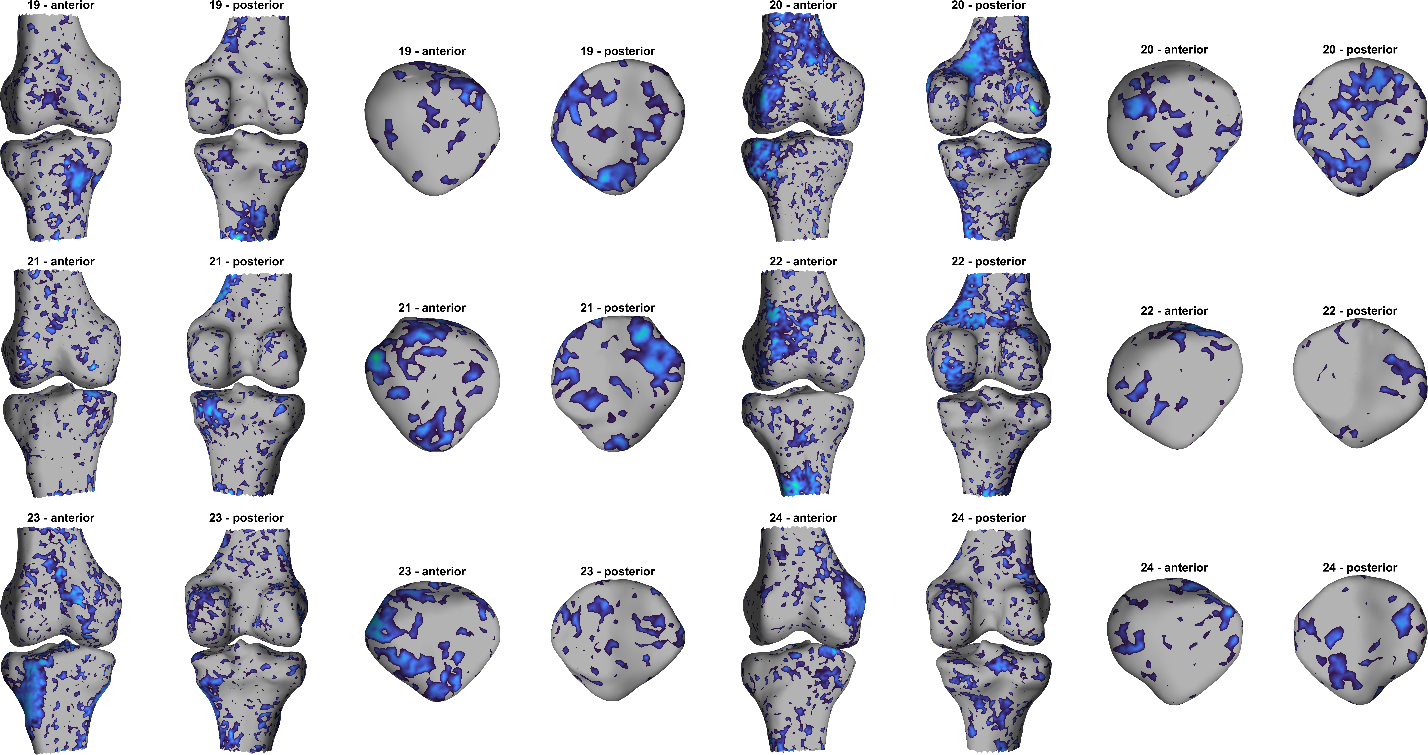  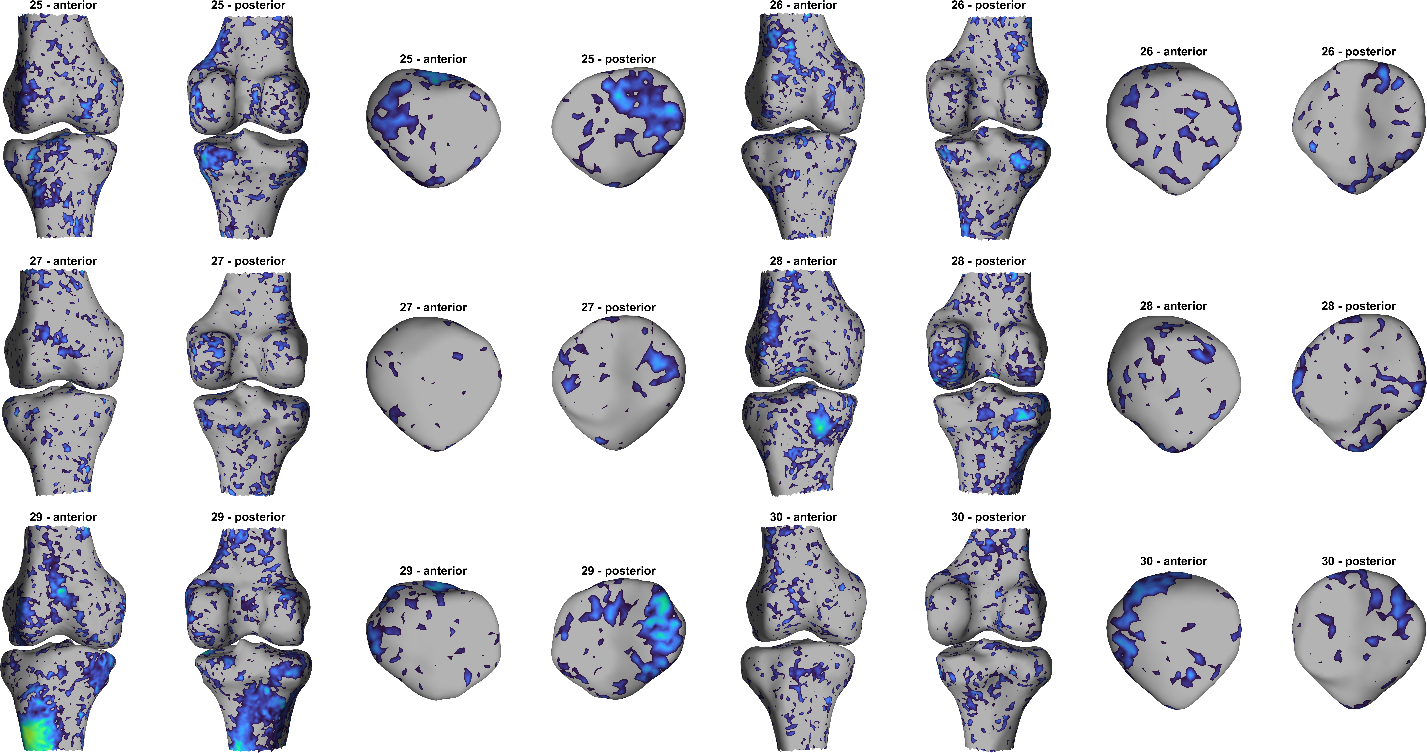  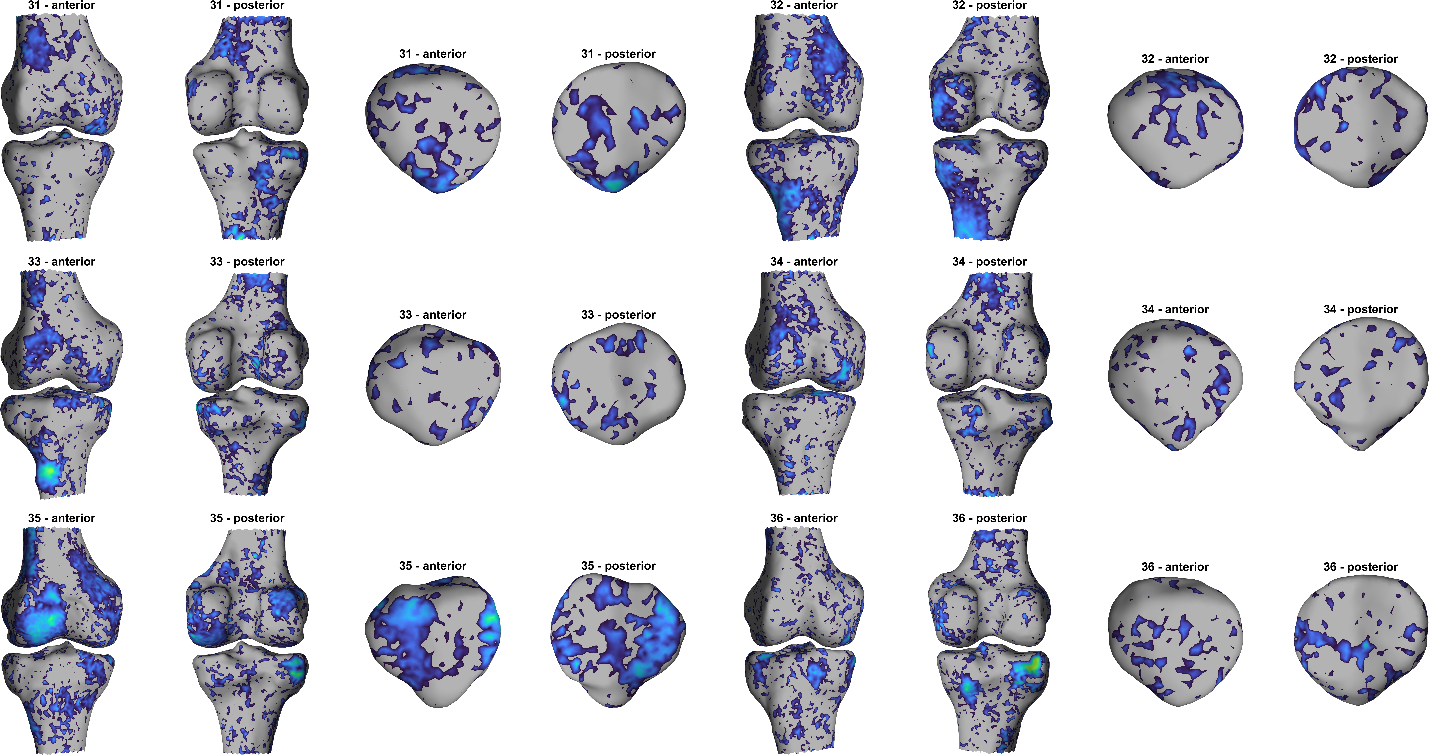  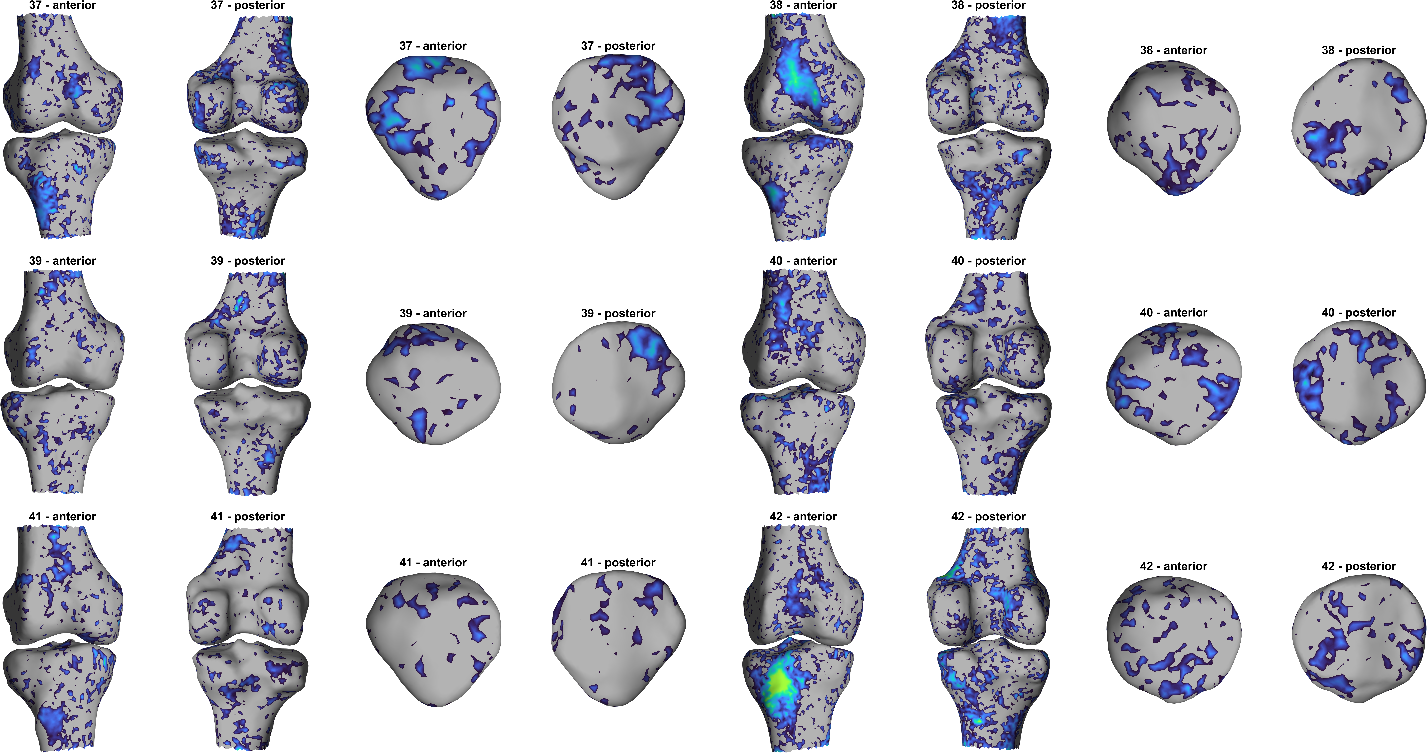  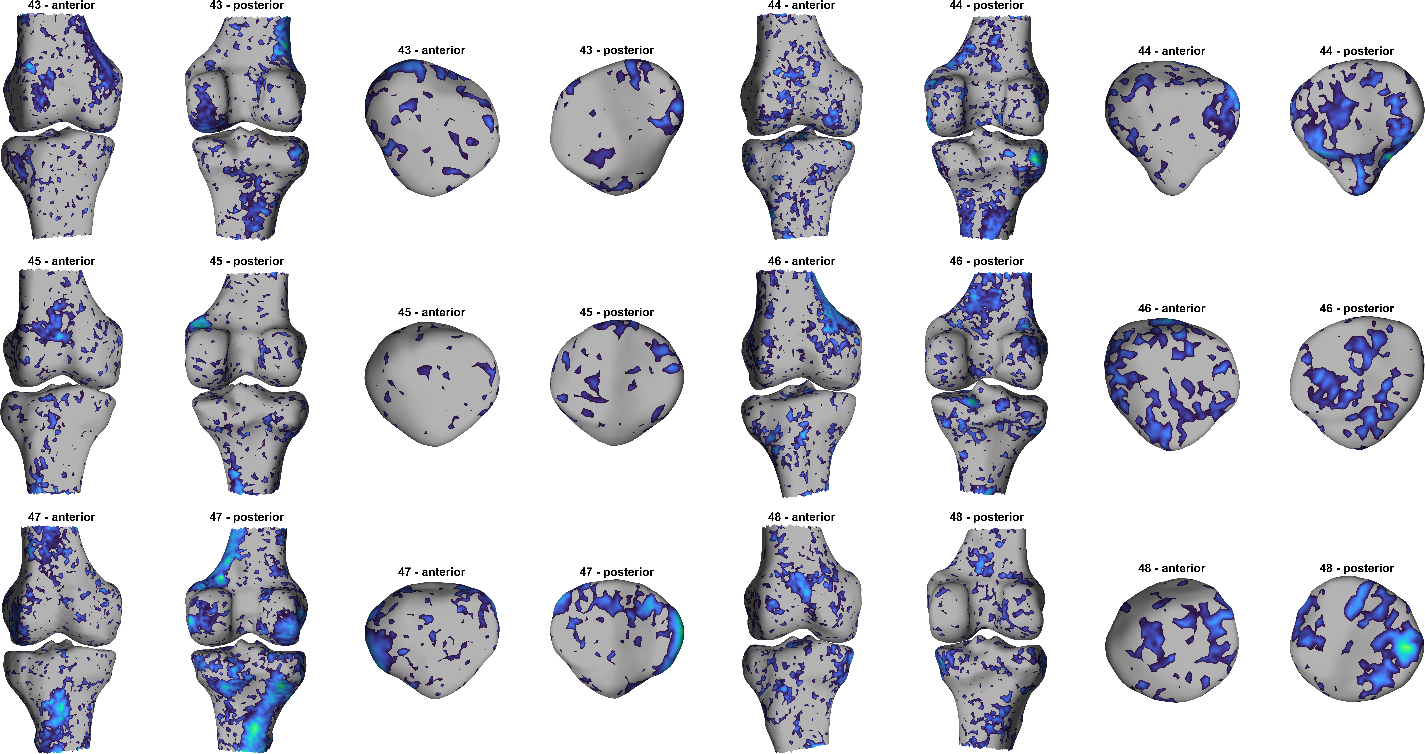  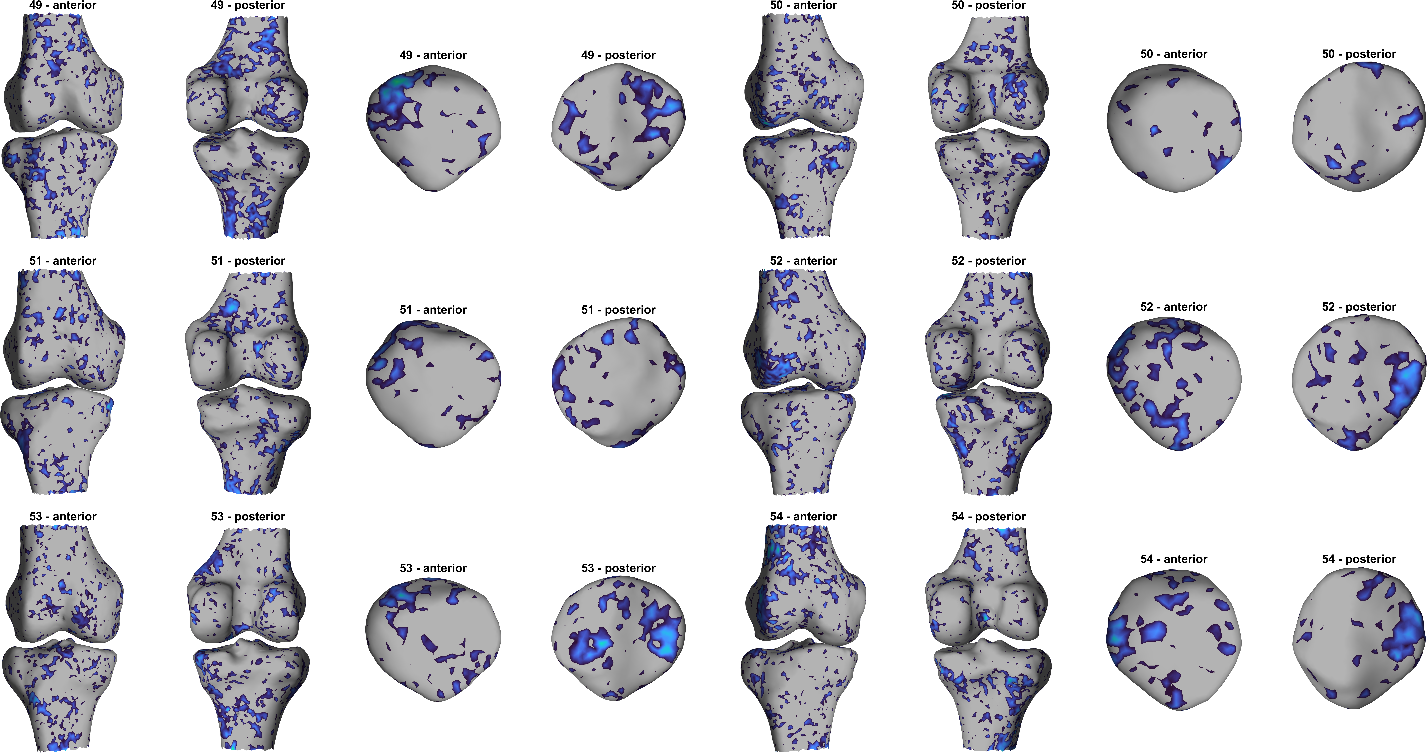  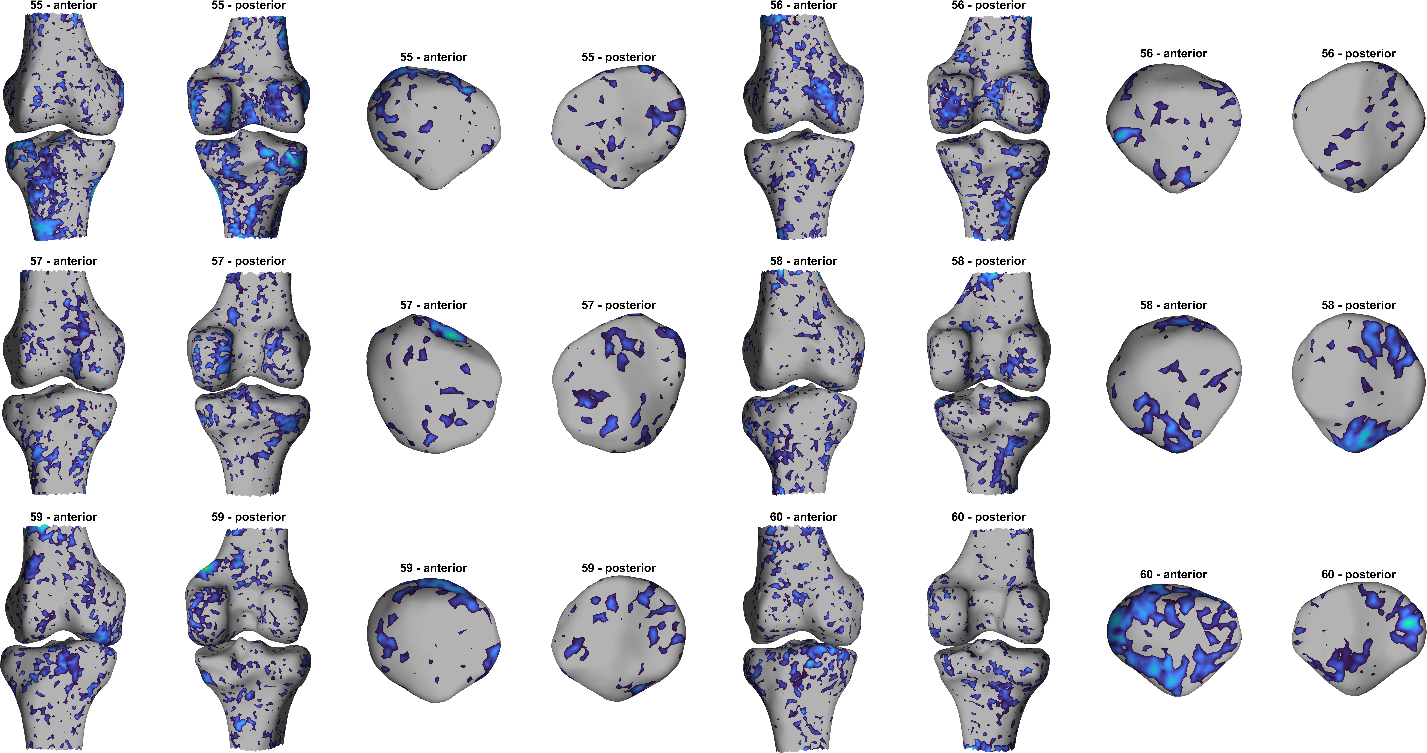  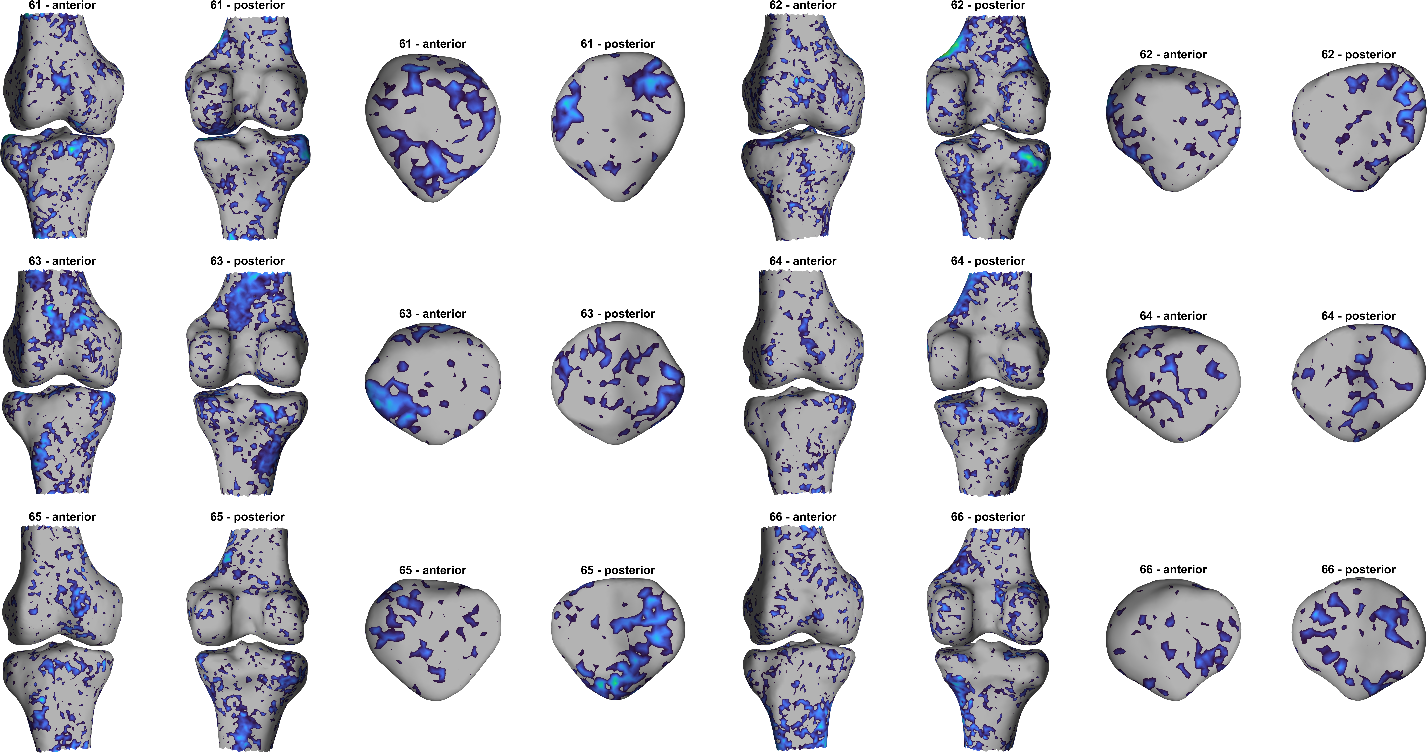  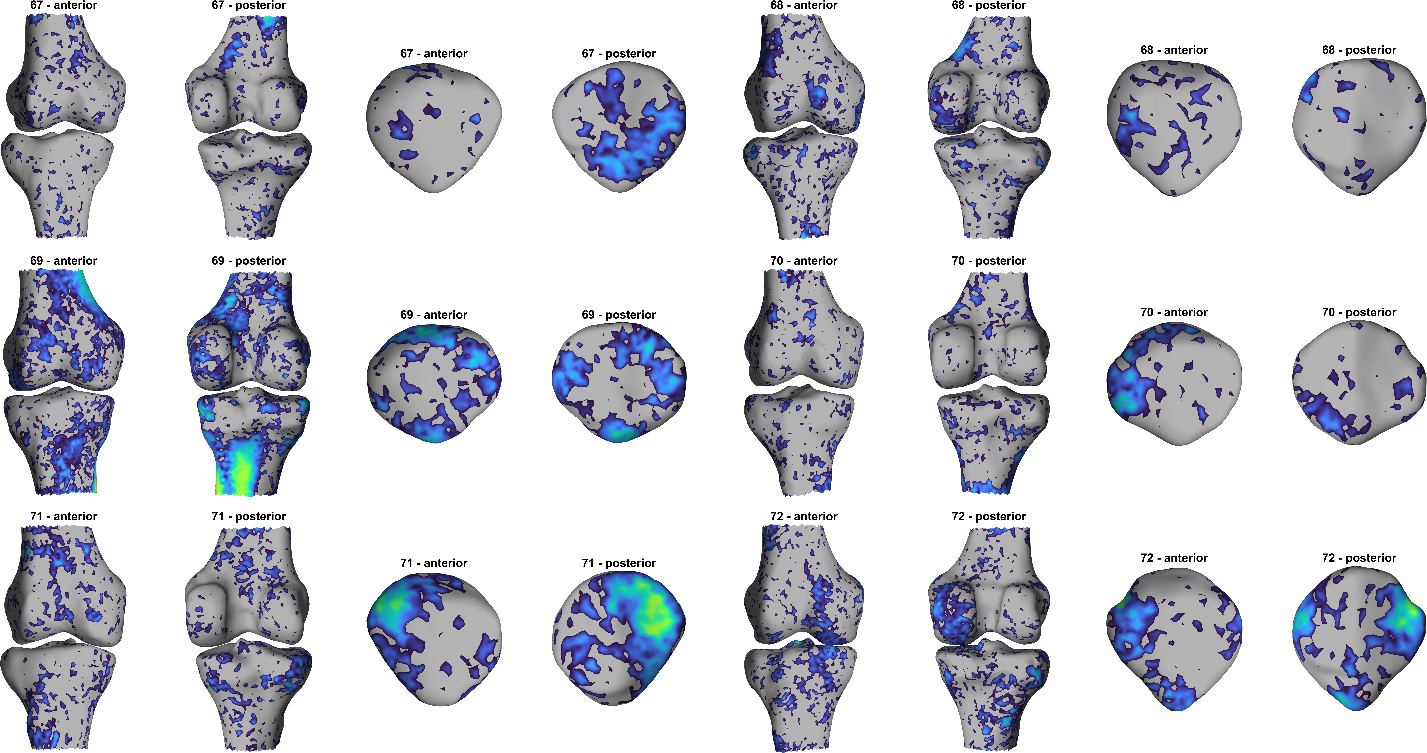  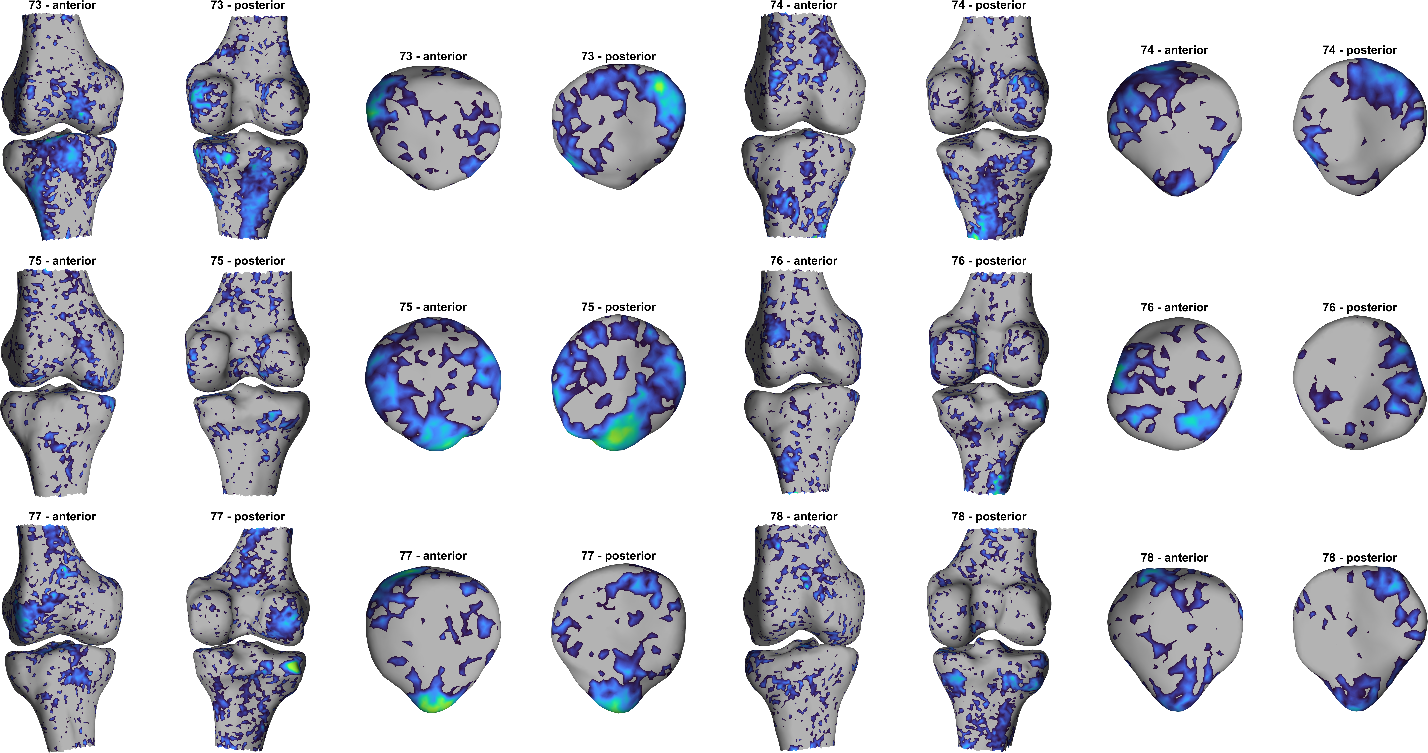  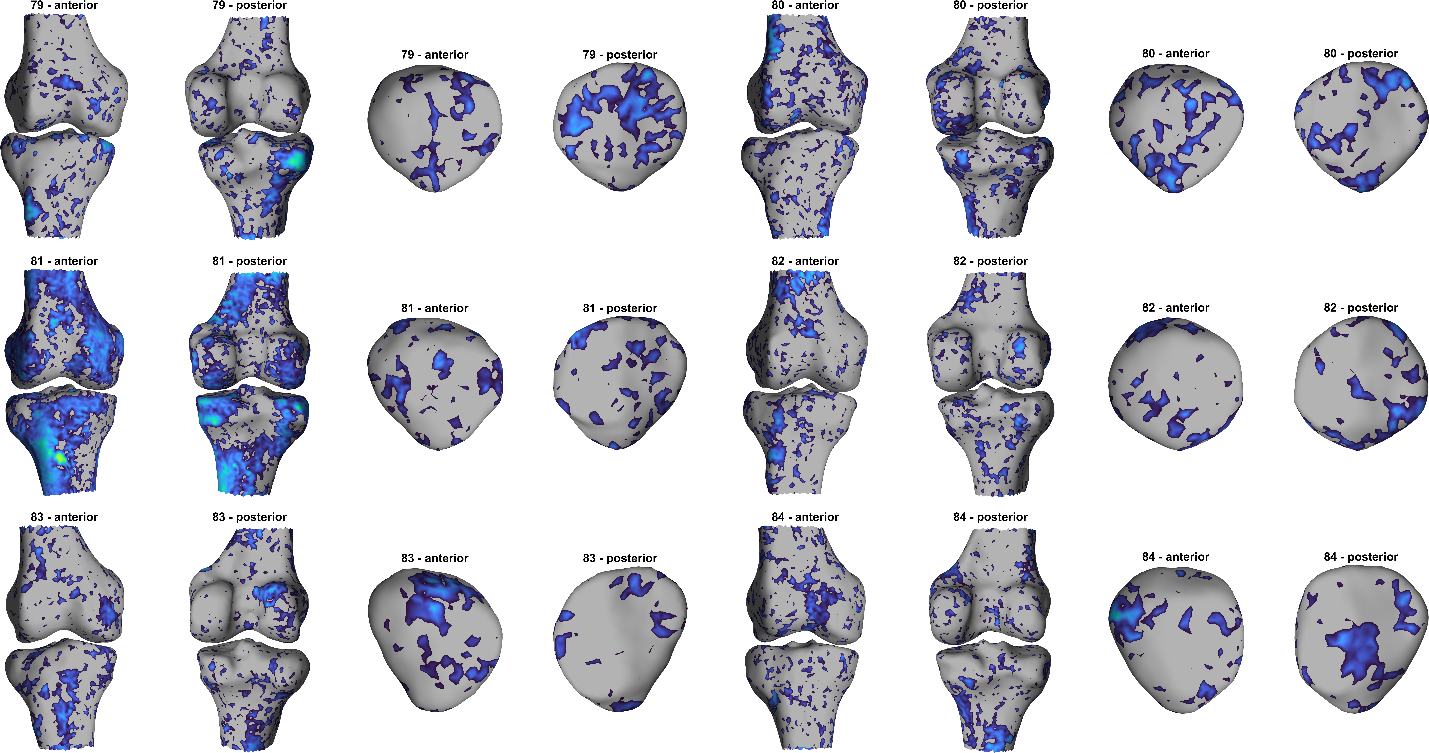  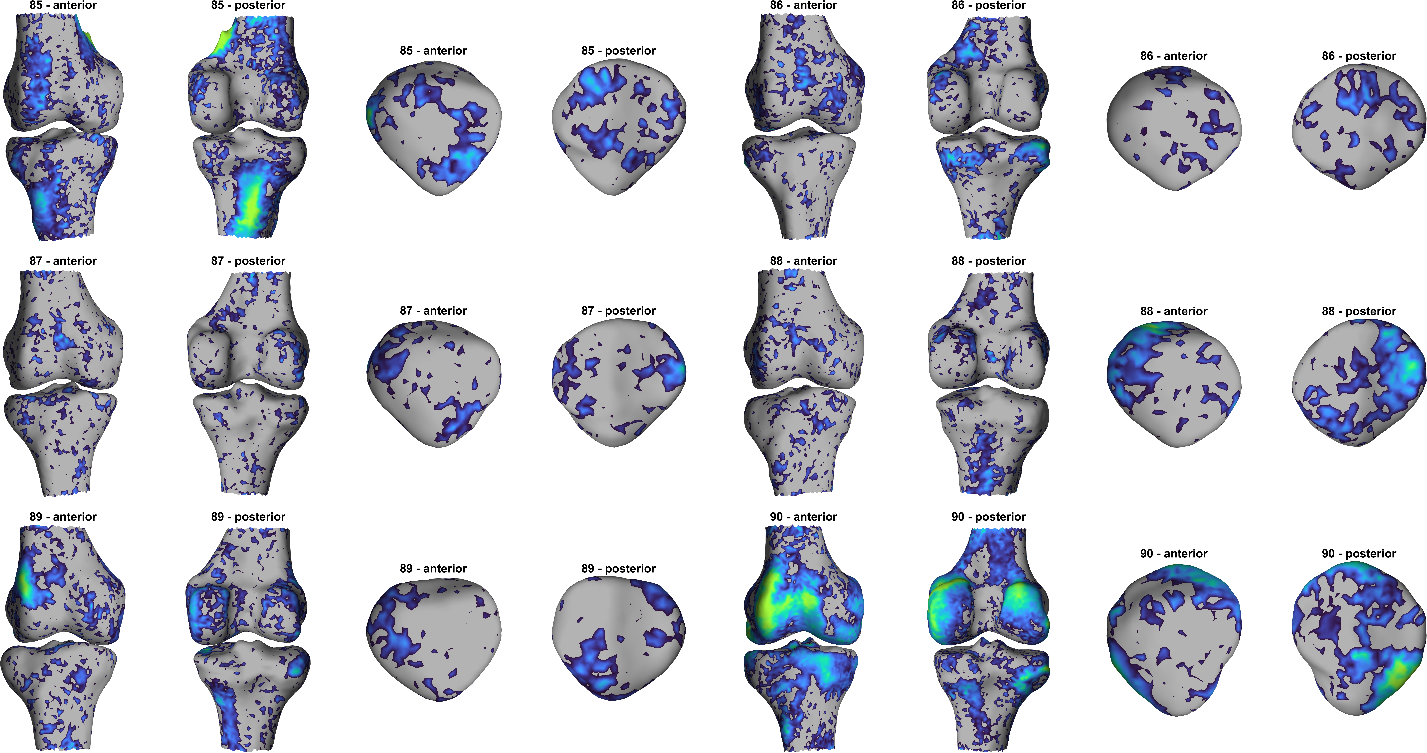  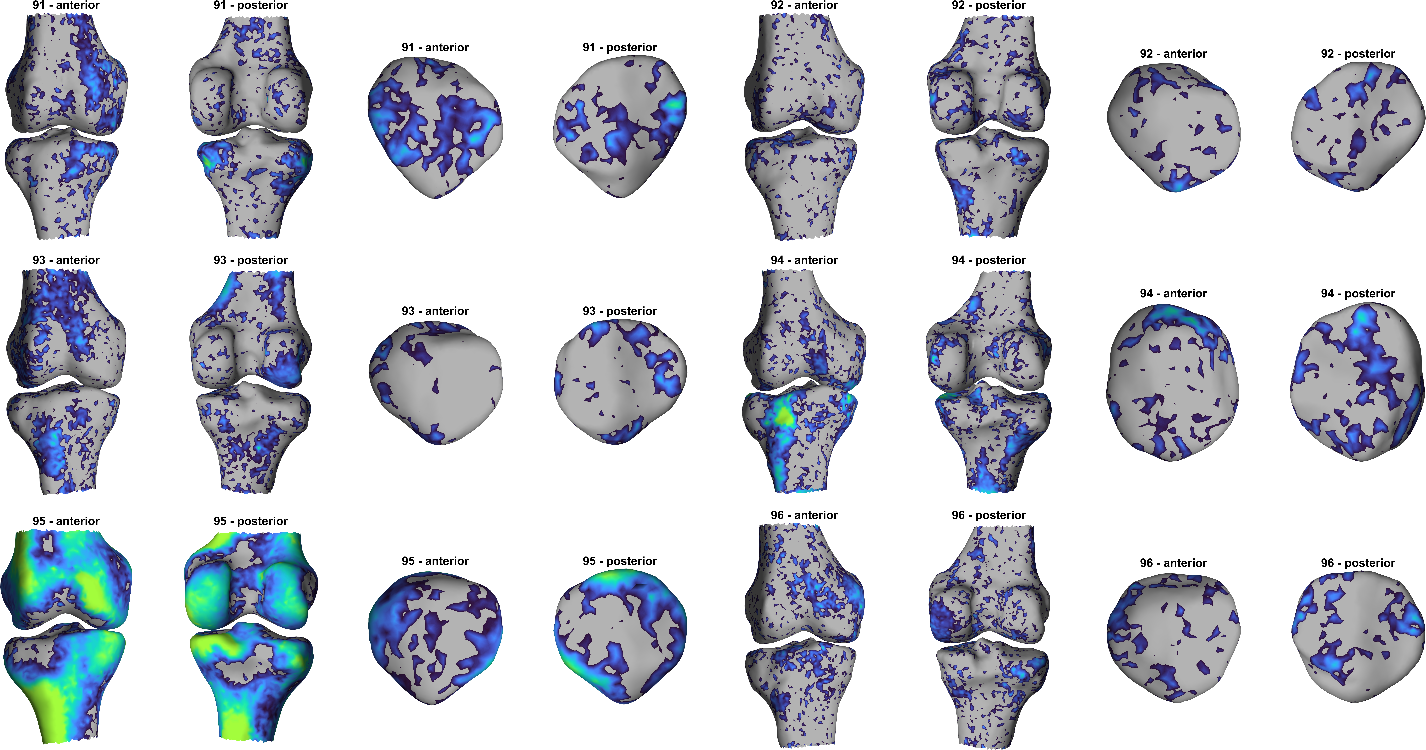  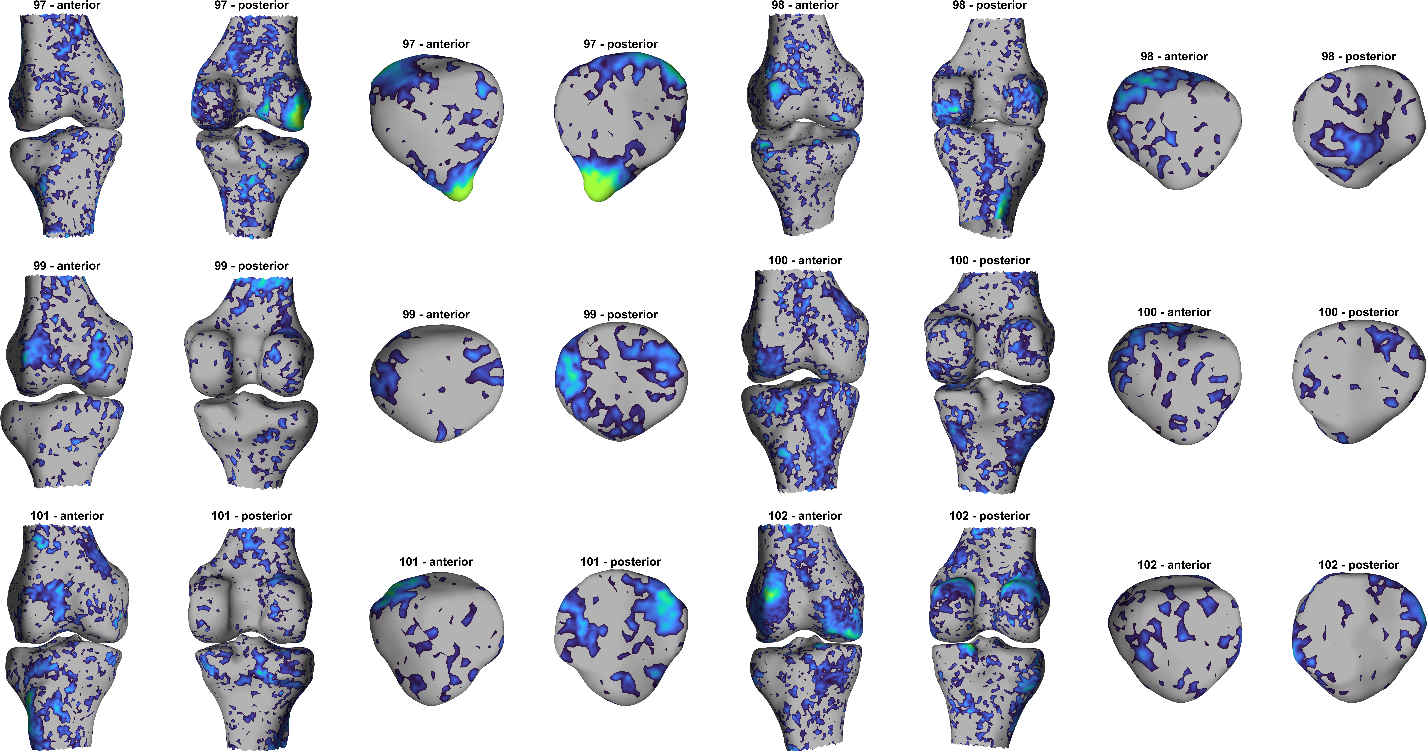  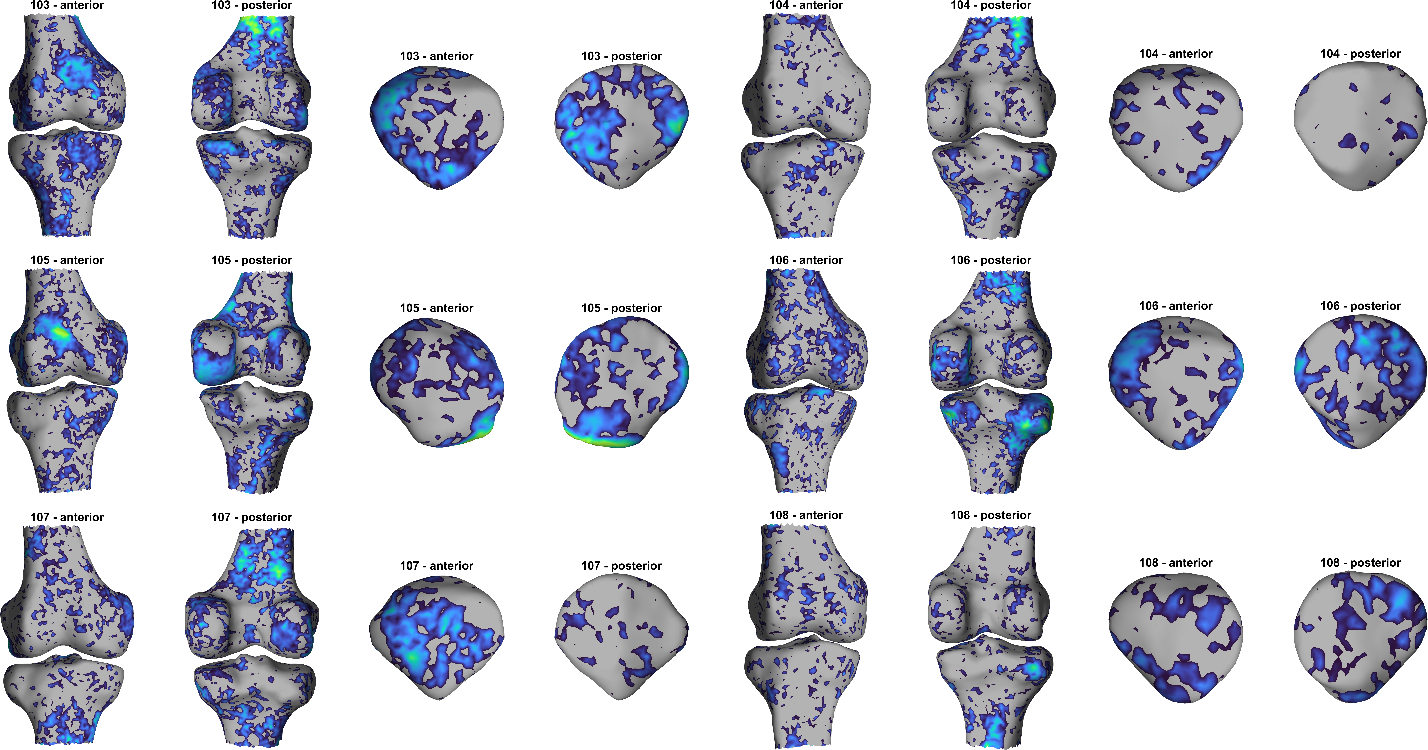  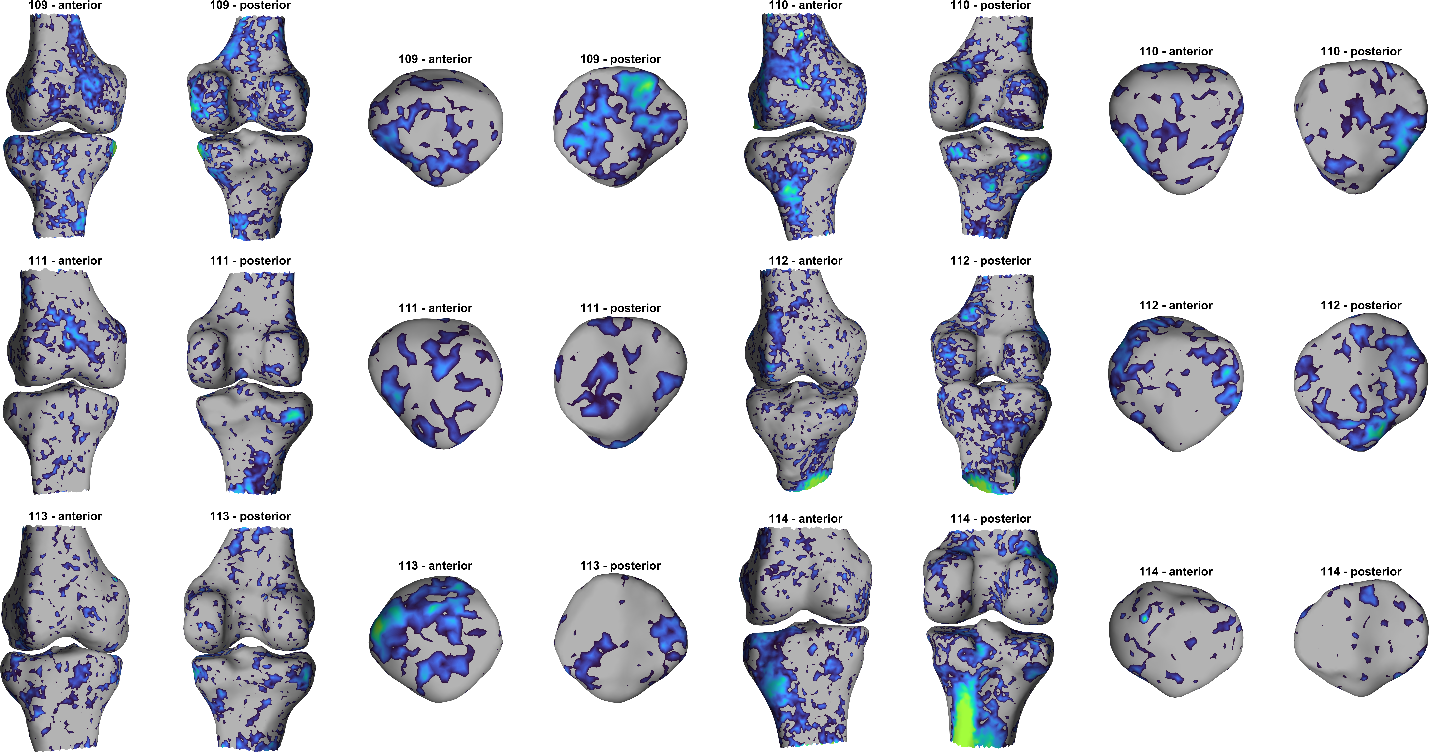  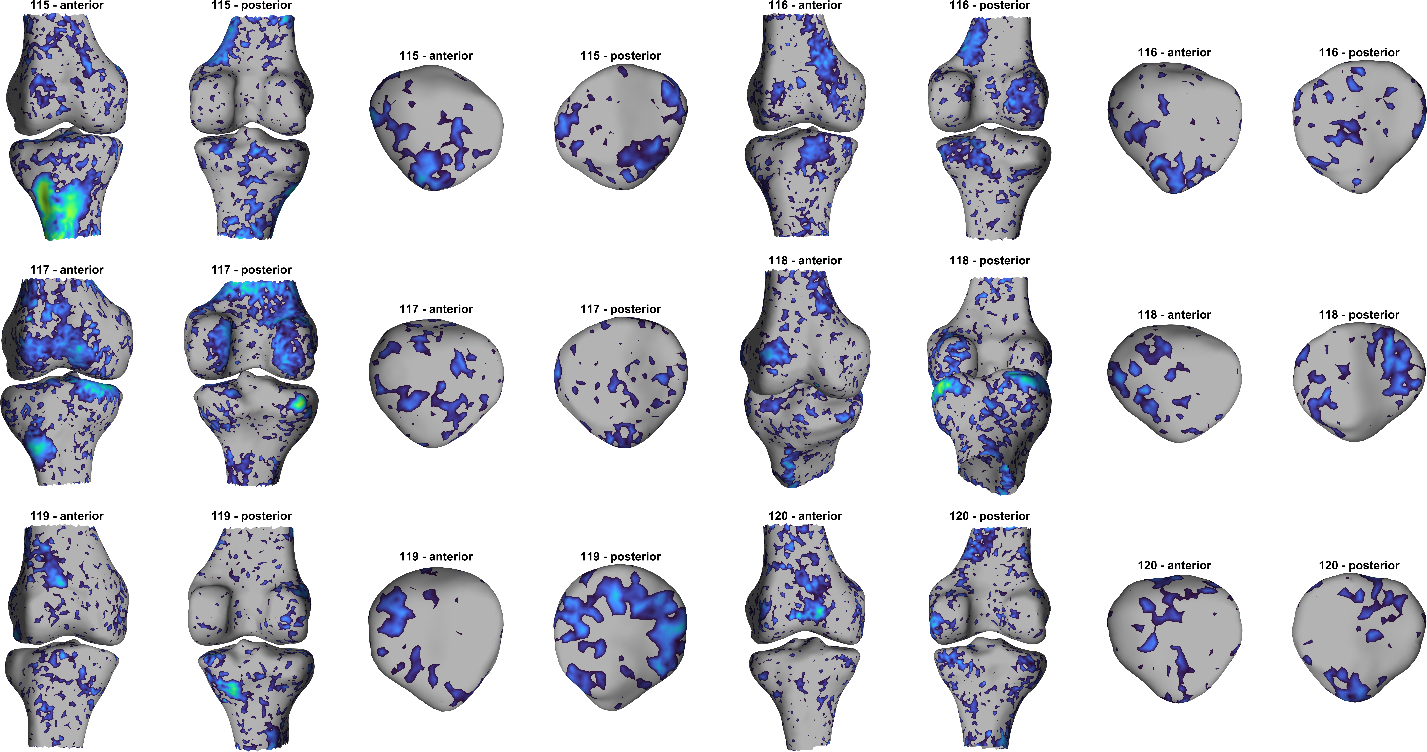  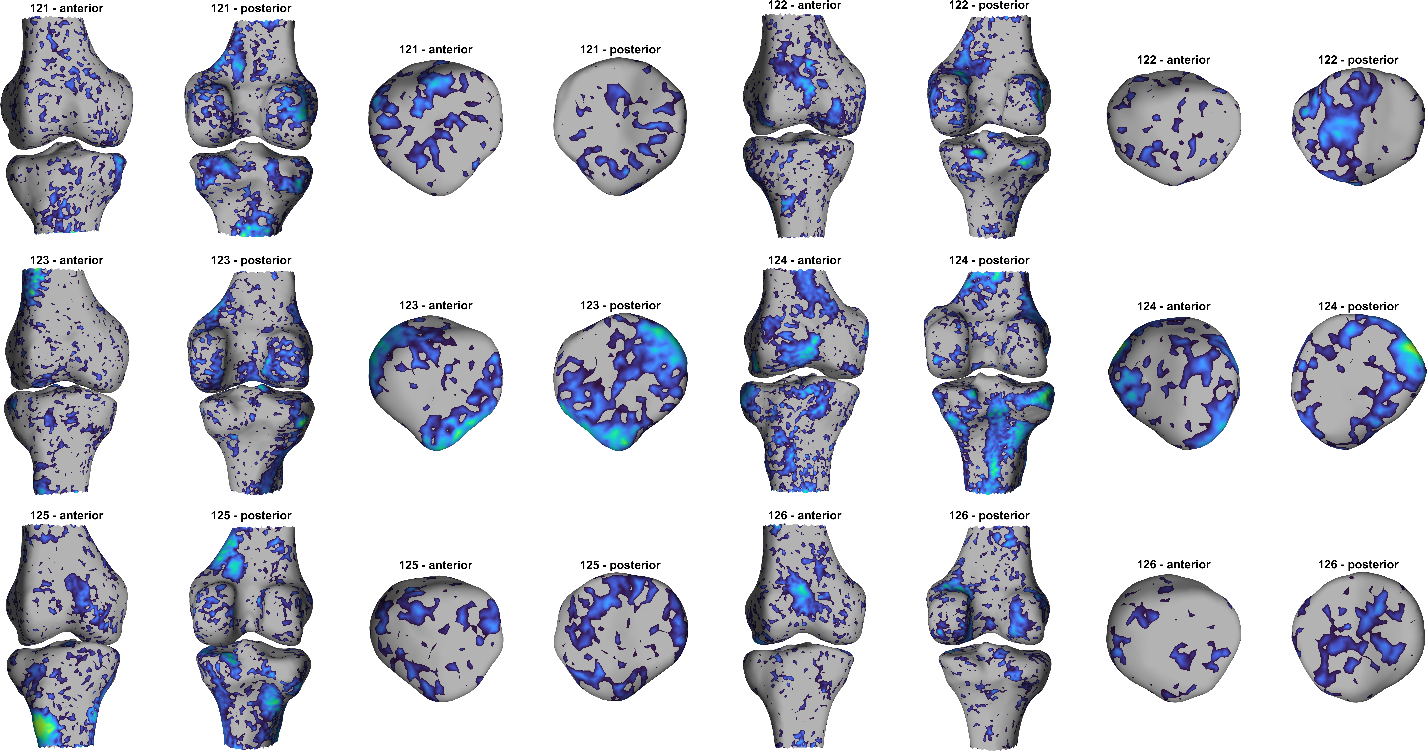  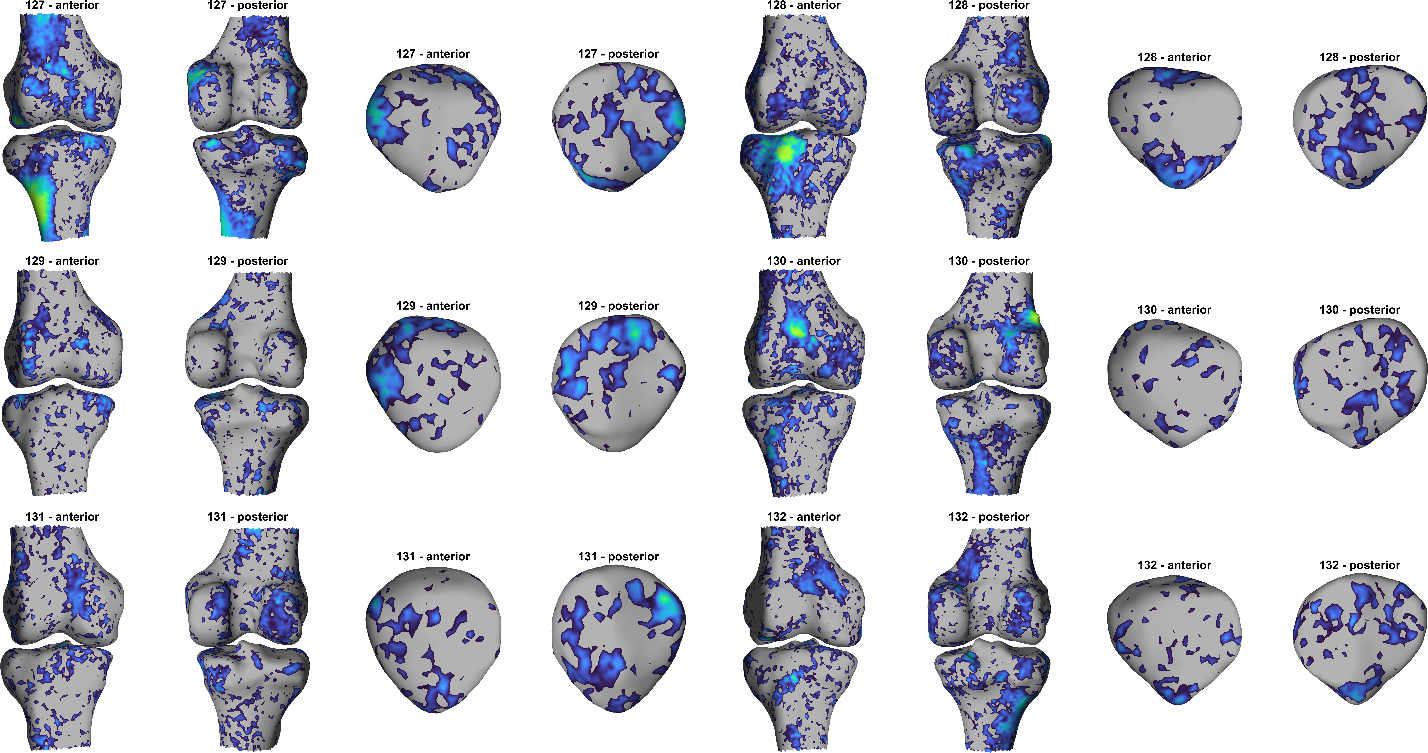  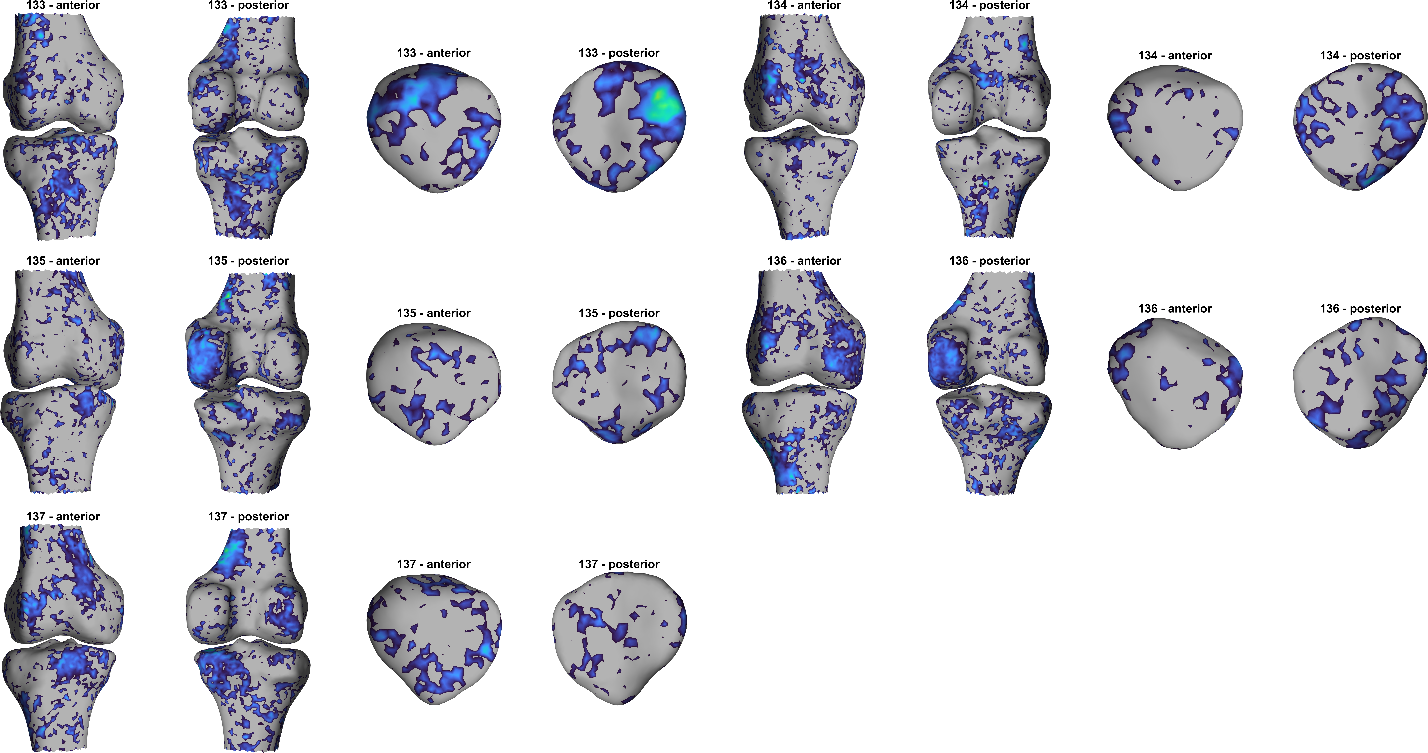 |
| --- |
| **Supplementary Material 4** heatmaps illustrating anterior and posterior left-right differences in bone morphology for knee pairs without trochlear dysplasia, displayed on the right knees. Deviations smaller than 1 mm are shown in grey. For each knee pair, the patella was scaled independently from the femur and tibia. |


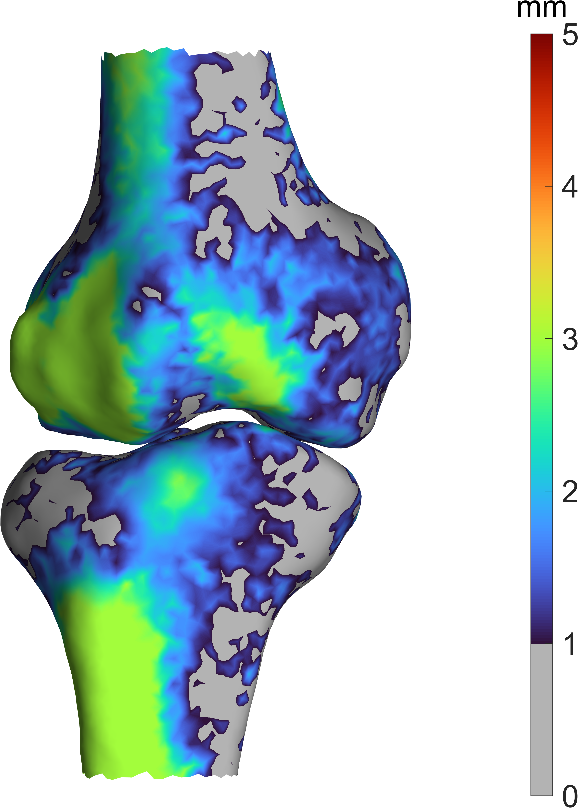


| 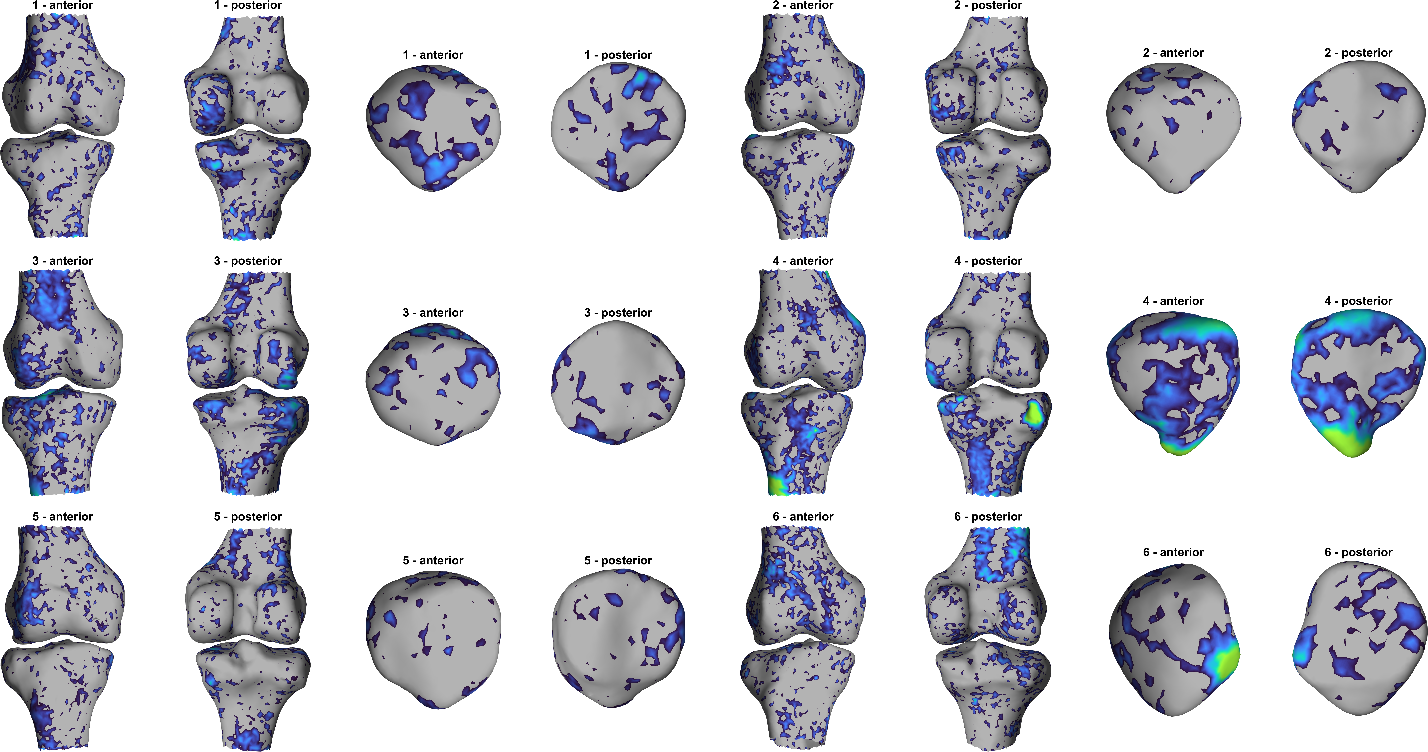  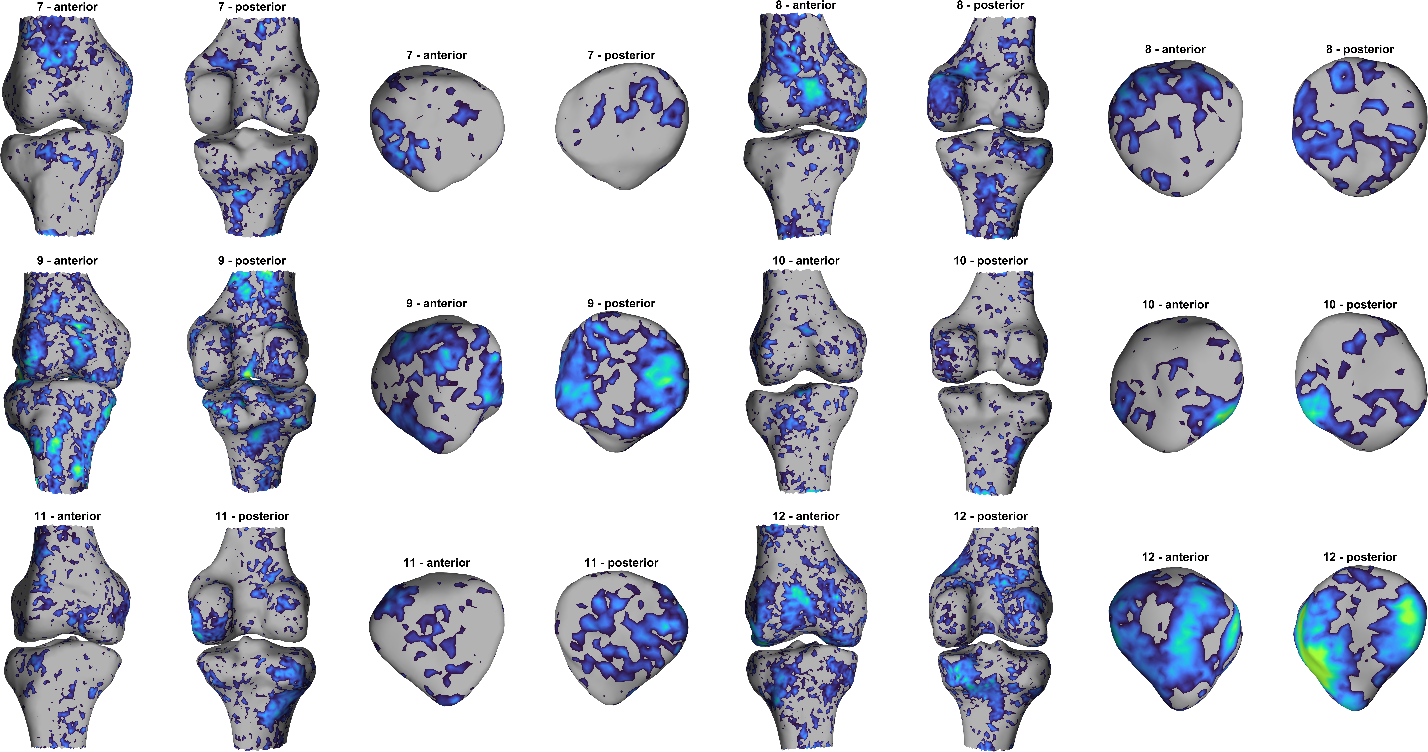  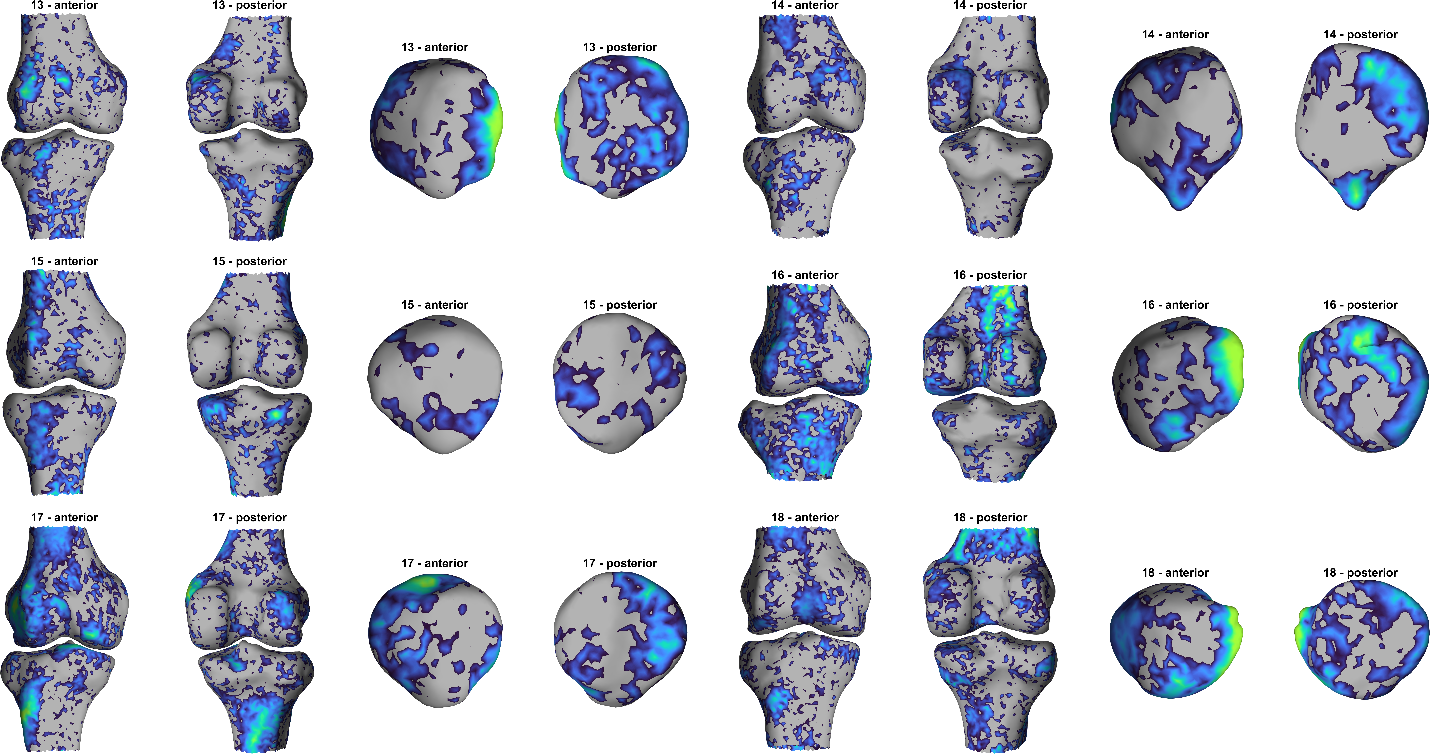  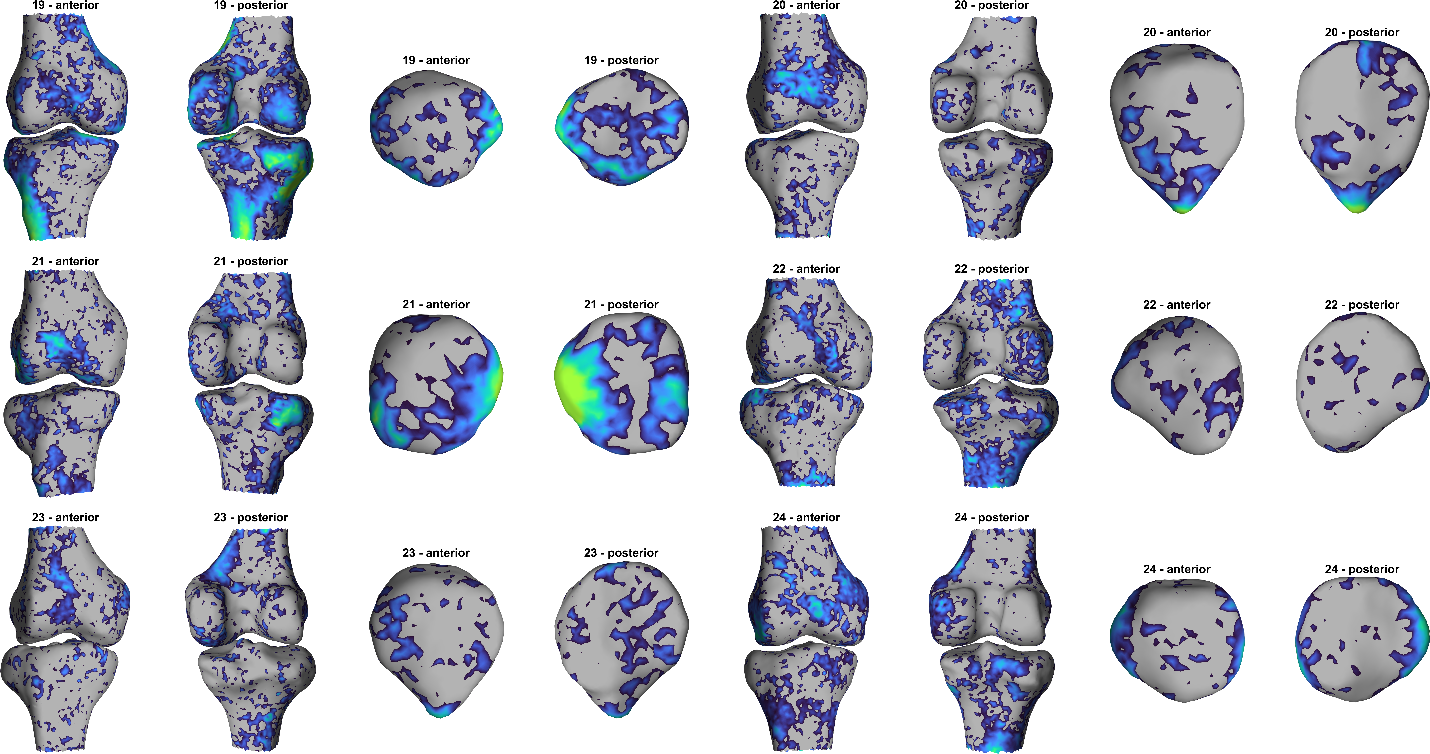  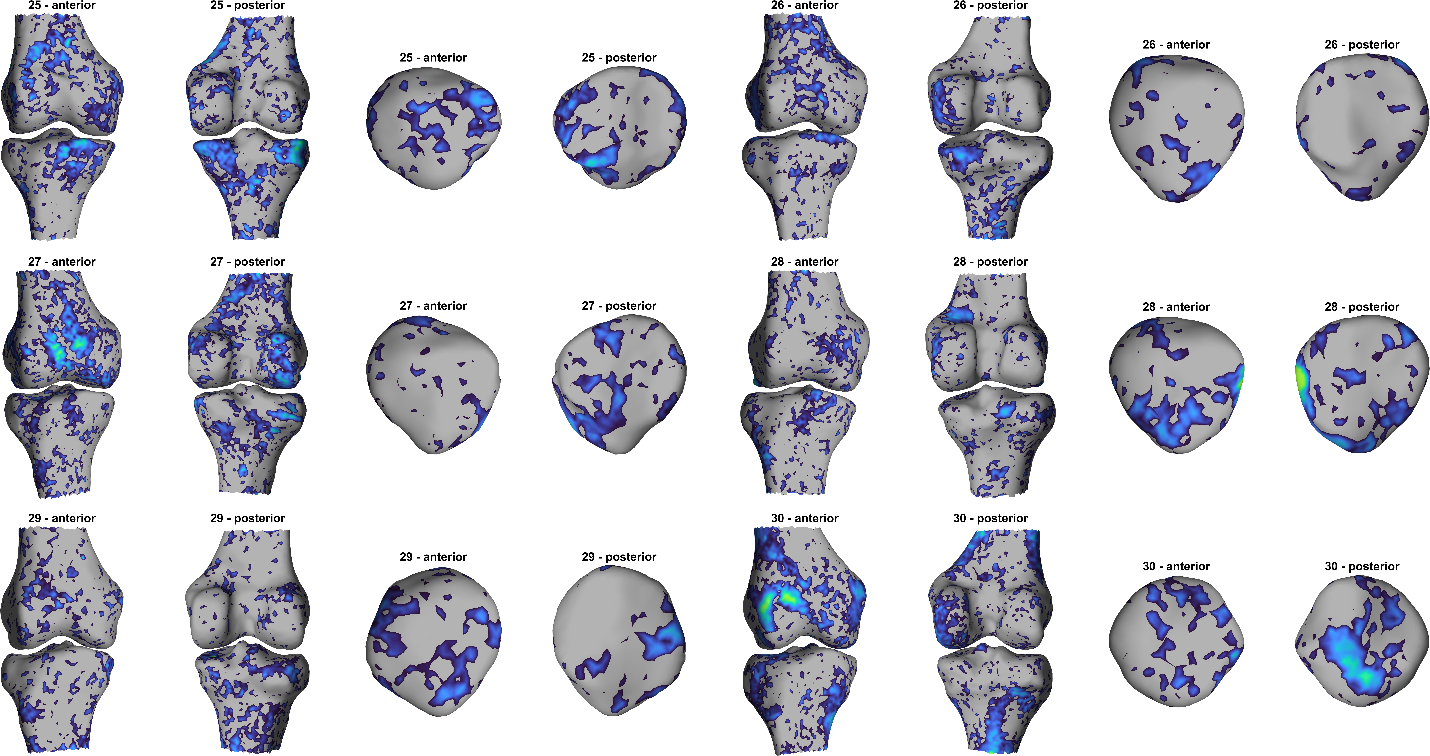  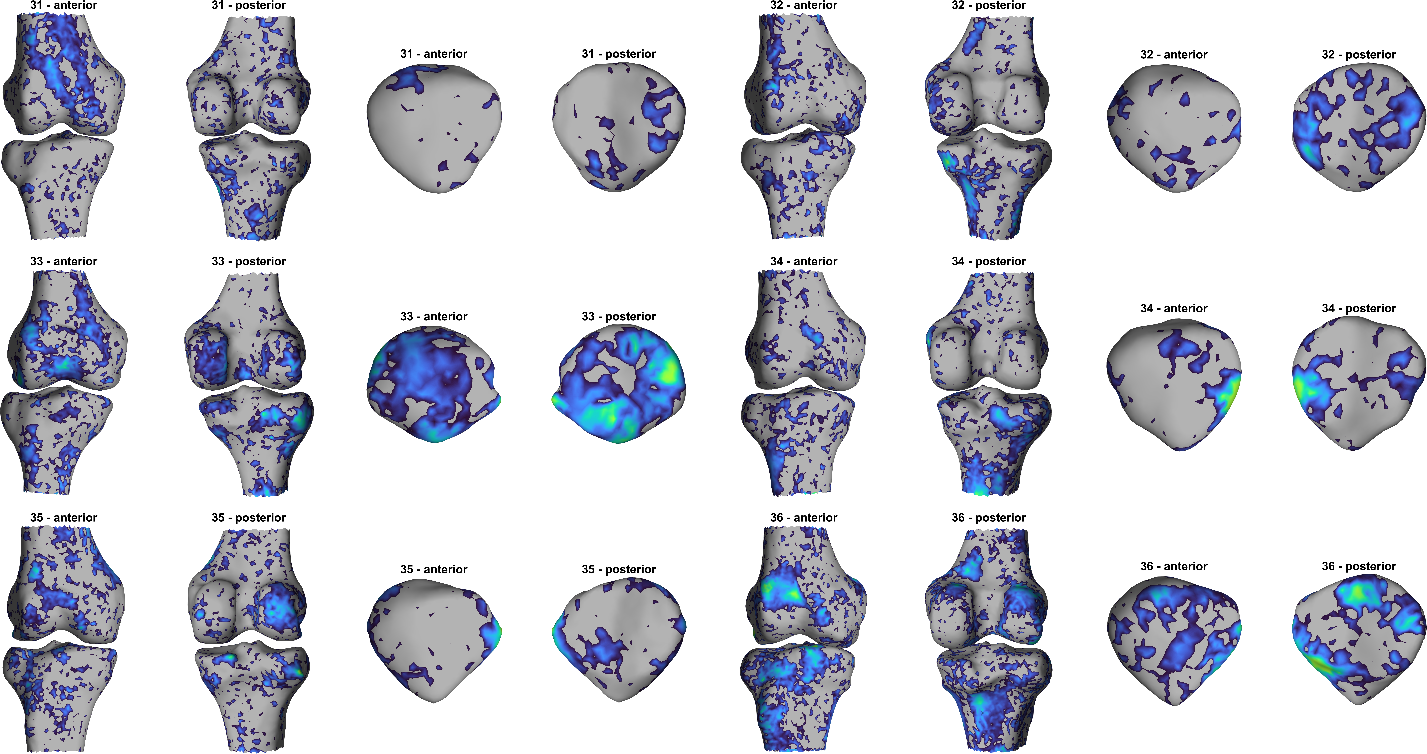  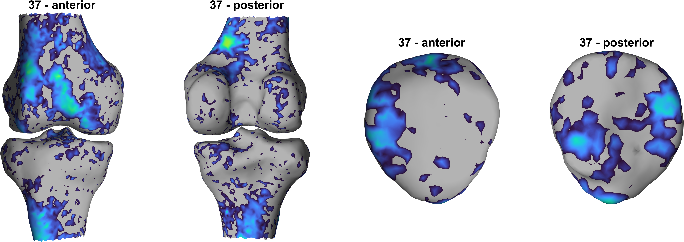 |
| --- |
| **Supplementary Material 5** heatmaps illustrating anterior and posterior left-right differences in bone morphology for knee pairs with low-grade trochlear dysplasia, displayed on the right knees. Deviations smaller than 1 mm are shown in grey, reflecting the measurement accuracy threshold. For each knee pair, the patella was scaled independently from the femur and tibia. |


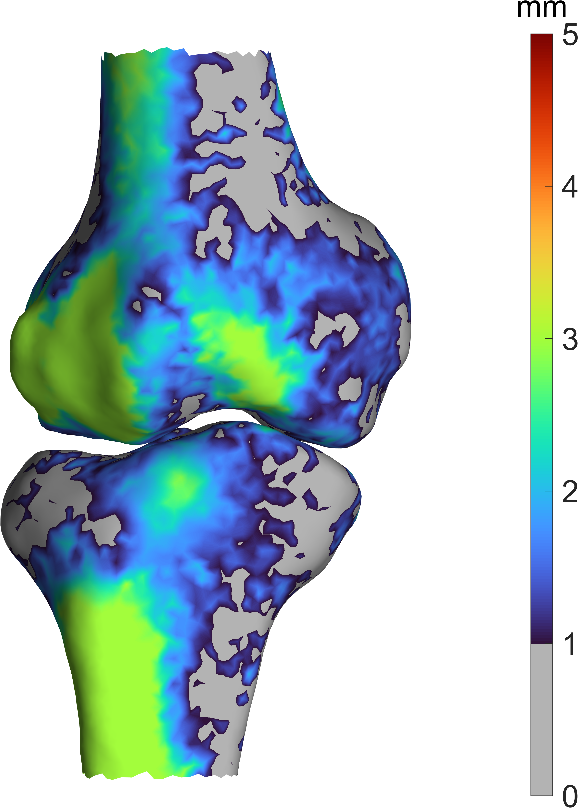


| 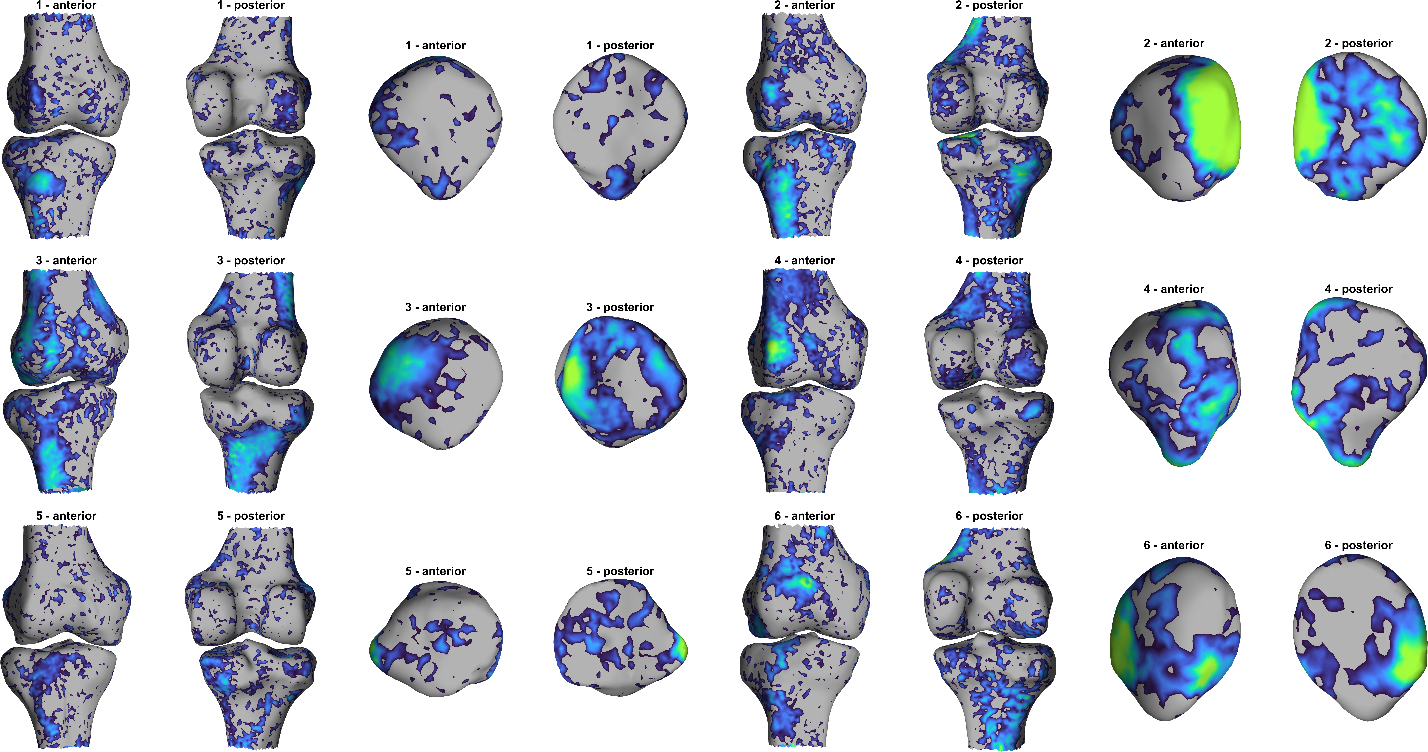  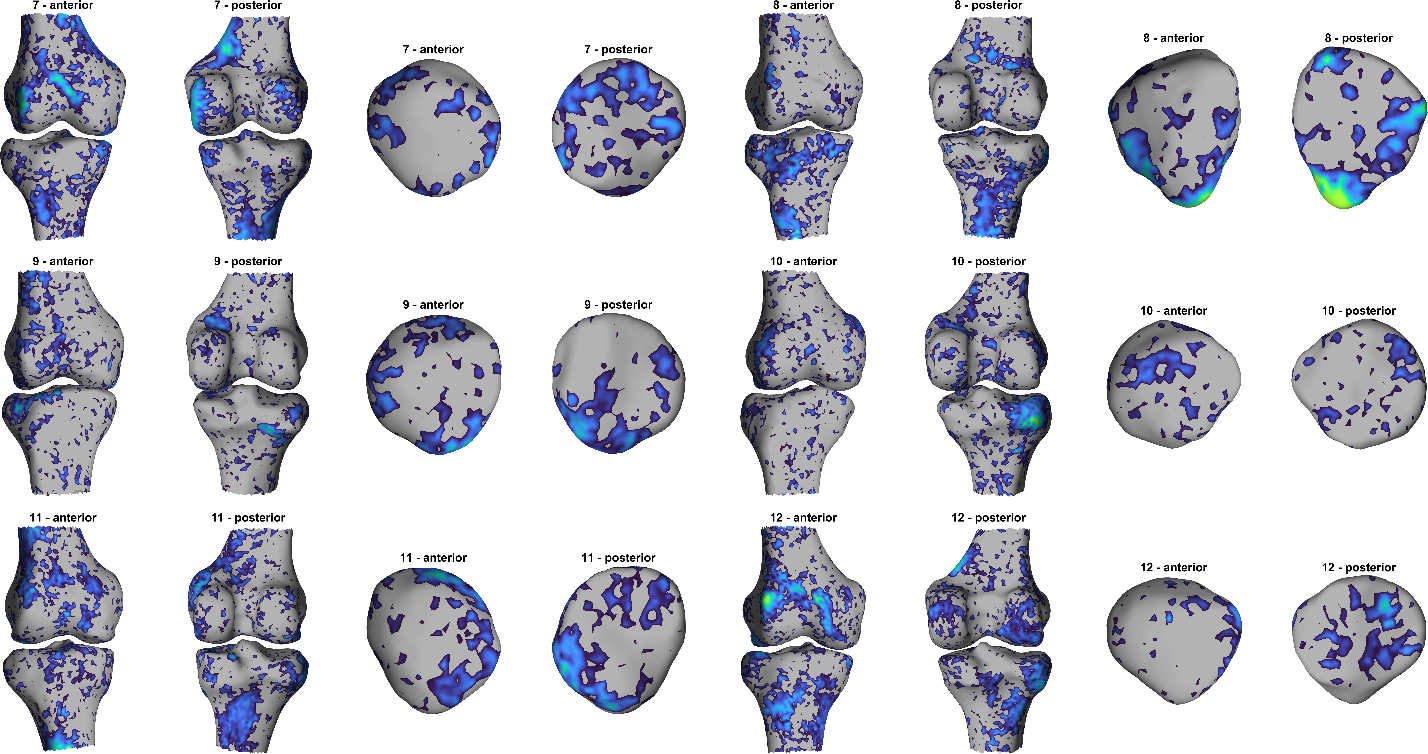  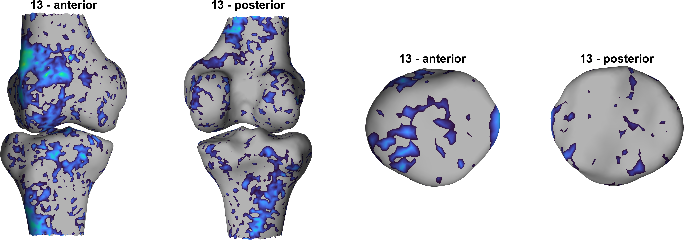 |
| --- |
| **Supplementary Material 6** heatmaps illustrating anterior and posterior left-right differences in bone morphology for knee pairs with high-grade trochlear dysplasia, displayed on the right knees. Deviations smaller than 1 mm are shown in grey, reflecting the measurement accuracy threshold. For each knee pair, the patella was scaled independently from the femur and tibia. |


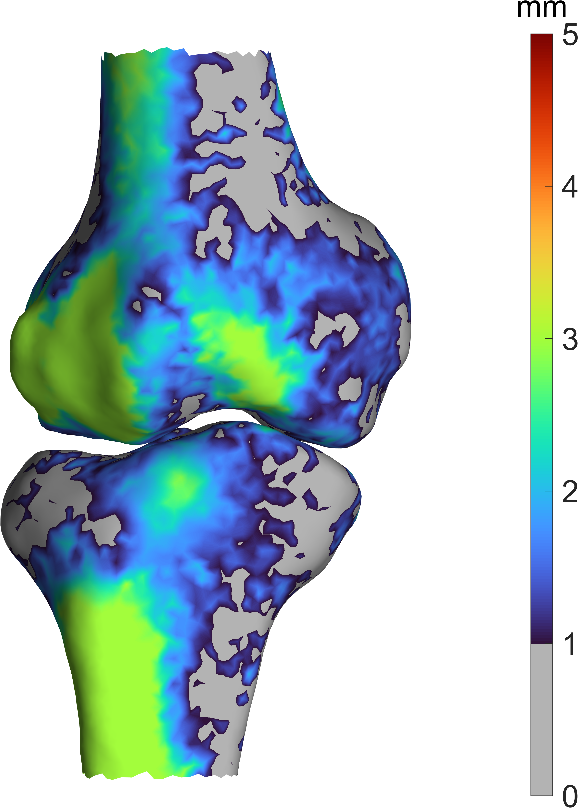

Supplement: Supplementary file 1 — Supplementary Material 1. [file 12891_2025_9272_MOESM1_ESM.docx]
